# TaSTP13 contributes to wheat susceptibility to stripe rust possibly by increasing cytoplasmic hexose concentration

**DOI:** 10.1186/s12870-020-2248-2

**Published:** 2020-01-30

**Authors:** Baoyu Huai, Qian Yang, Xiaobo Wei, Qinglin Pan, Zhensheng Kang, Jie Liu

**Affiliations:** 1 State Key Laboratory of Crop Stress Biology for Arid Areas, College of Plant Protection, Northwest A&F University, Yangling, China; 2 State Key Laboratory of Crop Stress Biology for Arid Areas, College of Life Sciences, Northwest A&F University, Yangling, China

**Keywords:** Wheat, Stripe rust, Sugar transporter, Susceptibility, VIGS

## Abstract

**Background:**

Biotrophic fungi make intimate contact with host cells to access nutrients. Sugar is considered as the main carbon sources absorbed from host cells by pathogens. Partition, exchanges and competition for sugar at plant-pathogen interfaces are controlled by sugar transporters. Previous studies have indicated that the leaf rust resistance (Lr) gene *Lr67*, a natural mutation of *TaSTP13* encoding a wheat sugar transport protein, confers partial resistance to all three wheat rust species and powdery mildew possibly due to weakened sugar transport activity of TaSTP13 by heterodimerization. However, one major problem that remains unresolved concerns whether *TaSTP13* participates in wheat susceptibility to rust and mildew.

**Results:**

In this study, expression of *TaSTP13* was highly induced in wheat leaves challenged by *Puccinia striiformis* f. sp. *tritici* (*Pst*) and certain abiotic treatments. TaSTP13 was localized in the plasma membrane and functioned as homooligomers. In addition, a functional domain for its transport activity was identified in yeast. Suppression of *TaSTP13* reduced wheat susceptibility to *Pst* by barley stripe mosaic virus-induced gene silencing (VIGS). While overexpression of *TaSTP13* promoted *Arabidopsis* susceptibility to powdery mildew and led to increased glucose accumulation in the leaves.

**Conclusions:**

These results indicate that *TaSTP13* is transcriptionally induced and contributes to wheat susceptibility to stripe rust, possibly by promoting cytoplasmic hexose accumulation for fungal sugar acquisition in wheat-*Pst* interactions.

## Background

During the Calvin cycle and gluconeogenesis processes, higher plants’ source leaves convert photosynthetically fixed CO_2_ into sugars, such as sucrose and its cleavage products, glucose, and fructose, which represent the central units for carbon metabolism, storage, and transport [1]. These sugars not only serve as essential substrates for fundamental plant growth processes but also are major energy sources and carbon precursors [2]. Additionally, sugars are pivotal signaling molecules that directly and indirectly regulate gene expression during the plant life cycle [2]. Sugar compartmentation mediated by sugar transporters is one of the major determinants for plant growth and various environmental stress responses [1, 3, 4].

Sucrose is a major long-distance translocation form of photoassimilate that is exported from the phloem to the apoplasm of sink tissues under hydrostatic pressure, and then subsequently imported into sink cells to sustain heterotrophic metabolism and growth by specific transport proteins [5]. This export step is mediated by SWEET proteins, sucrose transporters, or monosacharide transporters (if extracellular invertases are available) [3, 5]. These large sugar transporter families exert a fine-tuned regulation of sugar supply to satisfy different metabolic demands. Sugar transport proteins (STPs) are members of the monosaccharide transporters superfamily. There are 14 STPs found in *Arabidopsis* [1], 29 in rice [6], and 23 in barrel medic [7]. STPs are the best-characterized sugar transporter group in *Arabidopsis*, and all STP members have been identified in detail to date. All *Arabidopsis* STPs (AtSTPs) have been characterized as plasma membrane-localized H+/hexose symporters that show broad substrate specificity, with the exception of the non-functional AtSTP5 [8], AtSTP7 specific for L-arabinose and D-xylose [8], a glucose-specific transporter, AtSTP9 [9] and a galactose-specific transporter, AtSTP14 [10].

Pathogens are considered to be additional sinks that can lead to substantial changes in sugar partitioning within the plant. Uptake, exchanges, and competition for sugar at plant-pathogen interfaces are controlled by sugar transporters, and their regulation patterns are essential for determining the outcome of plant-pathogen interactions [7]. To date, there is increasing evidence that STPs play a central role in sugar translocation in pathogen-invaded plants. For example, in *Arabidopsis* infected with *Erysiphe cichoracearum*, up-regulation of *AtSTP4* and the cell-wall invertase, *AtβFRUCT1*, correlated with the transport of glucose into sink tissues [11]. In the grapevine’s response to infection by biotrophic pathogens, *VvHT5*, an *AtSTP13* ortholog, was transcriptionally activated and enhanced sink strength during the transition from source to sink [12]. Moreover, when *AtSTP13* was induced, it contributed to *Arabidopsis* resistance to grey mold disease by allowing living host cells to compete with *Botrytis cinerea* for apoplastic hexoses released by damaged tissues [13]. Additionally, AtSTP13 was phosphorylated by the BRASSINOSTEROID INSENSITIVE 1-associated receptor kinase 1 (BAK1) at threonine 485, which enhanced monosaccharide uptake activity to compete with bacteria for extracellular sugars, thus limiting the availability of extracellular sugar and depriving bacteria of an energy source, thereby restricting virulence factor delivery [14]. Clearly, apoplastic sugar control may constitute a host-defense strategy that limits a broad range of pathogens, including bacteria and fungi.

*Puccinia striiformis* f. sp. *tritici* (*Pst*), the causal agent of wheat stripe rust, is an obligate biotrophic fungus that acquires nutrients from host cells to survive. Sugar appears to be the main carbon source transported from host cells to pathogens [7]. However, little is known about the molecular mechanisms involved in the transfer of sugars or about the molecular responses induced in the host transport processes in response to pathogen invasion. Previous studies have found that Lr67 (a natural mutation of the sugar transporter TaSTP13) provides partial resistance to all three wheat rust species (i.e., stripe rust, leaf rust, and stem rust) and powdery mildew possibly due to a dominant-negative effect through heterodimerization with the functional transporters (TaSTP13) to reduce glucose uptake. Nevertheless, more details are still unclear. For example, whether is *TaSTP13* involved in wheat susceptibility to rust and mildew? Whether is reduce glucose uptake correlated with Lr67-mediated wheat resistance? In this study, the expression of *TaSTP13* homologues was significantly induced in wheat leaves challenged by the *Pst* pathotype, CYR31, and abiotic treatments. Subcellular localization analysis revealed that TaSTP13 is located in the plasma membrane. A critical functional domain for its transport activity was identified by heterologus mutant complementation in *Saccharomyces cerevisiae*. Knockdown of *TaSTP13* by the virus-induced gene-silencing (VIGS) system promoted wheat resistance to *Pst*. Transgenic *Arabidopsis* plants overexpressing *TaSTP13* showed enhanced susceptibility to powdery mildew and increased glucose accumulation in the leaves. Yeast two-hybrid (Y2H) and bimolecular fluorescence complementation (BiFC) validated oligomerization of TaSTP13. These results suggest that *TaSTP13* may contribute to wheat susceptibility to *Pst* by increasing fungal sugar supply.

## Results

### Cloning and sequence analysis of *TaSTP13*

The *TaSTP13* gene was amplified from the *Pst*-infected Su11 cDNA sample using reverse transcription polymerase chain reaction (RT-PCR). The obtained *TaSTP13* sequence was then blasted against the *T. aestivum* cv. Chinese Spring (CS) genome sequence. Results revealed that there are three copies located on chromosomes 4A, 4B, and 4D in the wheat genome. The putative coding sequences of these three copies only differ in 54 nucleotides, sharing 98.62% sequence identity (Additional file 1: Figure S1). Accordingly, the amino acid sequences deduced from the three copies share 99.42% sequence identity (Additional file 2: Figure S2). And also, they are identical to the LR67sus protein form the wheat cultivar Thatcher in amino acid sequence, while they are different from the Lr67res protein form Thatcher RL6077 [15] at the two key amino acid residues: Gly144 and Val387 (Additional file 3: Figure S3). Multi-alignment of TaSTP13, LR67sus and LR67res was shown in Additional file 3: Figure S3.

The open reading frame (ORF) of *TaSTP13* consists of 1545 nucleotides, which encodes a peptide of 514 amino acids with a calculated molecular weight of 56.71 kDa. The TaSTP13 protein sequence was used as a query sequence to search the most up-to-date databases. By doing so, the highest similarity STPs from other plant species were found. The protein sequence had 99.32% identity with HvSTP13 from barley (IPK Barlex accession No: HORVU4Hr1G067450); 97.49% identity with an STP13-like protein from *Brachypodium distachyon*, *BdMST4* (GenBank accession No: XP_003558480.1); and 96.81% identity with OsMST4 (an STP13-like protein) from rice (GenBank accession No: XP_015630449.1). The phylogenetic tree of TaSTP13 with these homologous proteins, as well as the STP family members from *Arabidopsis*, was constructed. These results revealed that all STP13-like proteins form a unique clade that is different from other *Arabidopsis* STP family members in the phylogenetic tree (Additional file 4: Figure S4), indicating that STP13 is conserved during plant evolution. In addition, TaSTP13 was most closely related to STP13-like proteins from monocotyledons, compared to those from dicotyledons (Additional file 4: Figure S4). These results suggest that TaSTP13 may also be an STP13-like protein.

### Expression pattern of TaSTP13 under different treatments

**Fig. 1:**
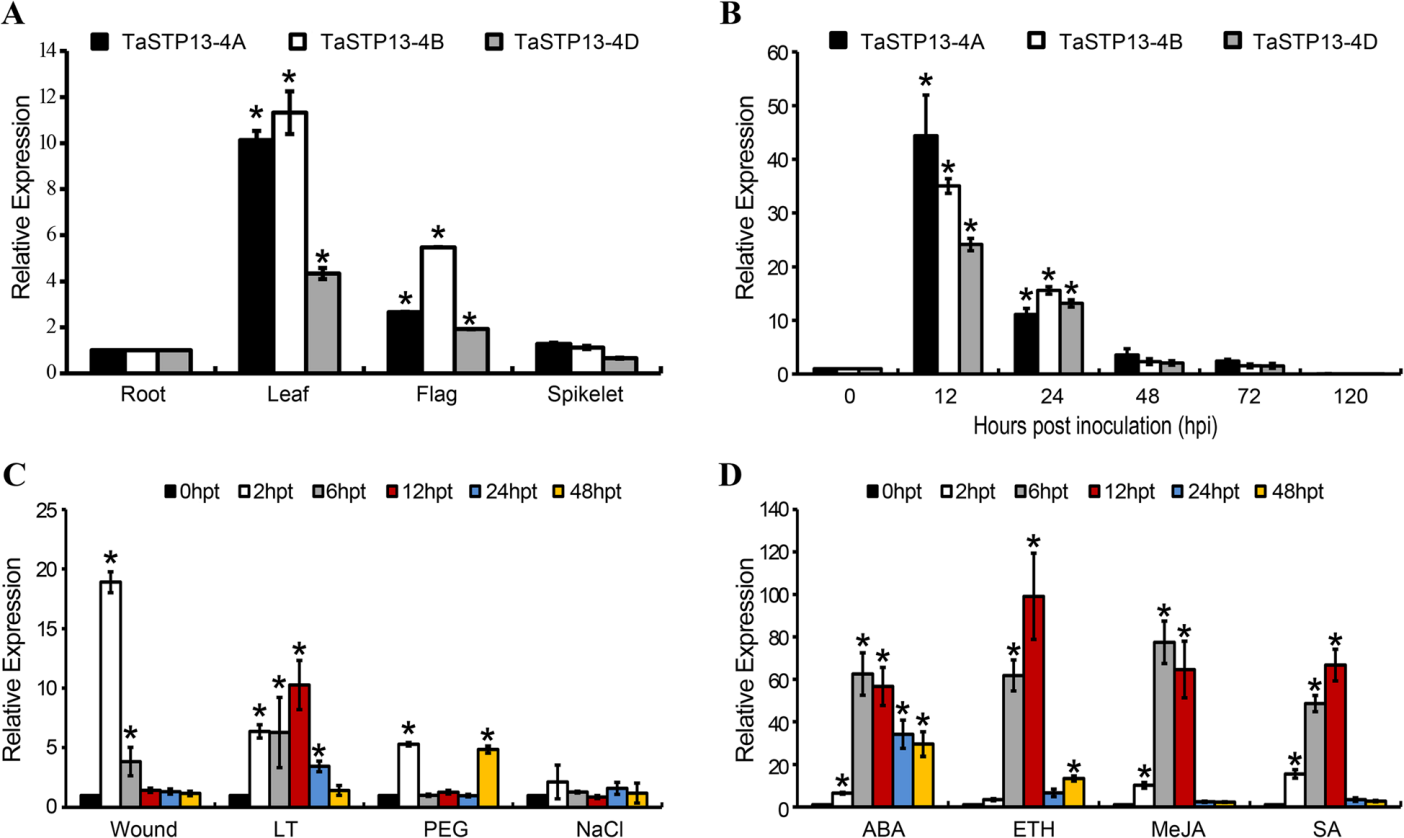
Expression patterns of the three TaSTP13 copies. **a** Transcript profile of *TaSTP13* in different wheat tissues. Samples were collected from roots, leaves, flags and spikelet. The level of *TaSTP13* was defined as 1 in root tissue; levels in other tissues are presented as relative ratios. **b** Transcriptional changes of the three *TaSTP13* copies induced by *Pst* infection. Wheat leaves infected with CYR31 were collected at 0, 12, 24, 48, 72, and 120 hpi. Transcript profile of *TaSTP13-4A* in response to abiotic stress (**c**) and exogenous hormones (**d**). Wheat leaves were sampled at 0, 2, 6, 12, 24 and 48 hpt. Expression levels were normalized to *TaEF-1a*. The relative expression of *TaSTP13* was calculated using the comparative threshold method (2^–ΔΔ*C*^_T_). Asterisks indicate a significant difference (*P* < 0.01) according to Student’s *t*-test. Bars indicate the mean ± SD of three independent biological replicates. ABA, abscisic acid; ETH, ethylene; MeJA, methyl jasmonate; SA, salicylic acid; LT, low tempreture; PEG; polyethyleneglycol 6000

To determine the expression patterns of *TaSTP13* in different wheat tissues, quantitative real-time PCR (qRT-PCR) was performed with specific primers (Additional file 9: Table S2). Consistent with transcriptomic data from the WheatExp (https://wheat.pw.usda.gov/WheatExp/), qRT-PCR revealed that the three copies of *TaSTP13* are ubiquitously expressed in roots, leaves, flags, and spikelets (Fig. 1a). Their transcript abundance in green leaves was predominantly higher than that in other tissue (Fig. 1a). Additionally, *TaSTP13* was also expressed in stems and flowers (data not shown). Transcriptome analysis of *Pst*-infected wheat leaves suggest that the *TaSTP13* transcript level was up-regulated [16, 17]. To further confirm the increased expression characteristics, the transcript level of *TaSTP13* was measured using qRT-PCR. The results illustrated that the transcript levels of all three *TaSTP13* copies sharply increased by more than 25- and 10-fold at 12 and 24 h post-inoculation (hpi) with CYR31, respectively (Fig. 1b).

Additionally, because STPs are intensely regulated by many abiotic factors [1], the *TaSTP13* response to various environmental stressors was determined. The expression levels of the three copies in wheat leaves treated with wounding, low temperature (LT), polyethyleneglycol (PEG) 6000, and NaCl were measured by qRT-PCR. Results revealed that *TaSTP13* expression was up-regulated at 2 h, and then decreased after the wounding treatment (Fig. 1c, Additional file 5: Figure S5A, C). During low temperature stress, transcript levels of *TaSTP13* were keenly induced 2–24 h post treatment (hpt) and peaked at 12 hpt (Fig. 1c, Additional file 5: Figure S5A, C). *TaSTP13* transcripts were up-regulated at 2 h after PEG6000 treatment, then subsequently decreased from 6 to 24 hpt and increased again at 48 hpt (Fig. 1c, Additional file 5: Figure S5A, C). Under the NaCl treatment, changes were not observed in transcript abundance of *TaSTP13* compared to the control (Fig. 1c, Additional file 5: Figure S5A, C).

Additionally, *TaSTP13* transcript levels were assayed in wheat leaves after treatment with exogenous plant hormones abscisic acid (ABA), ethylene (ETH), methyl jasmonate (MeJA), and salicylic acid (SA). After hormone treatment, *TaSTP13* expression was induced, peaking at 12 hpt (Fig. 1d, Additional file 5: Figure S5B, D). Taken together, these results clearly indicate that the transcription of *TaSTP13* responds to abiotic factors, hormone elicitors, and *Pst*.

### TaSTP13 localization in the plasma membrane

**Fig. 2:**
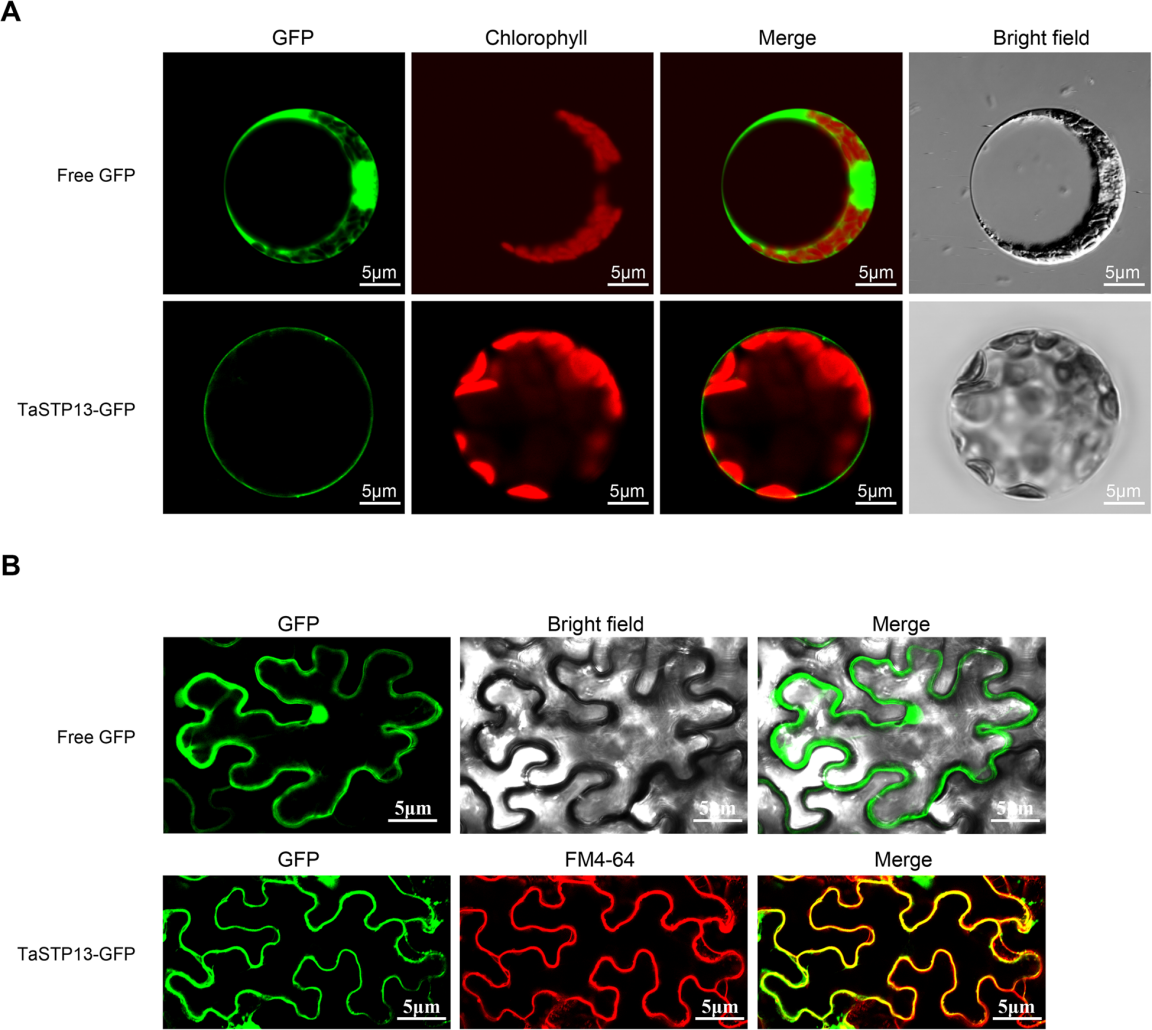
Subcellular localization of TaSTP13 in plants. **a** Transient expression of free GFP (control) and TaSTP13-GFP under control of the 35S promoter in wheat leaf protoplasts. **b** GFP and TaSTP13-GFP fusion proteins expressed in *N. benthamiana* by transient agro-infiltration assays. The All signals were monitored using confocal microscopy. FM4–64 was used to stain the plasma membrane. GFP fluorescence is in green; red fluorescence is from FM4–64 to label the plasma membrane or auto-fluorescence from chloroplasts. Merged images are depicted here. Bright field images show the equivalent field observed under white light. Comparable expression and localization patterns were observed in three independent biological replicates

To examine the subcellular localization of TaSTP13, TaSTP13- GFP translational fusions driven by the cauliflower mosaic virus (CaMV) 35S promoter were constructed. High fluorescence of free GFP (control) was found throughout the cell, including the nucleus (Fig. 2a-b). The TaSTP13-GFP fusions were predominantly distributed at the periphery (plasma membrane) of transformed wheat leaf protoplasts. GFP fluorescence indicated plasma membrane association of TaSTP13-GFP fusion proteins (Fig. 2a). Similarly, in *Nicotiana benthamiana* epidermal cells, GFP fluorescence signals of TaSTP13-GFP coincided with the FM4–64-labeled plasma membrane (Fig. 2b). Thus, it is likely that TaSTP13 is a plasma membrane-localized sugar transporter.

### Functional domain analysis of TaSTP13

**Fig. 3:**
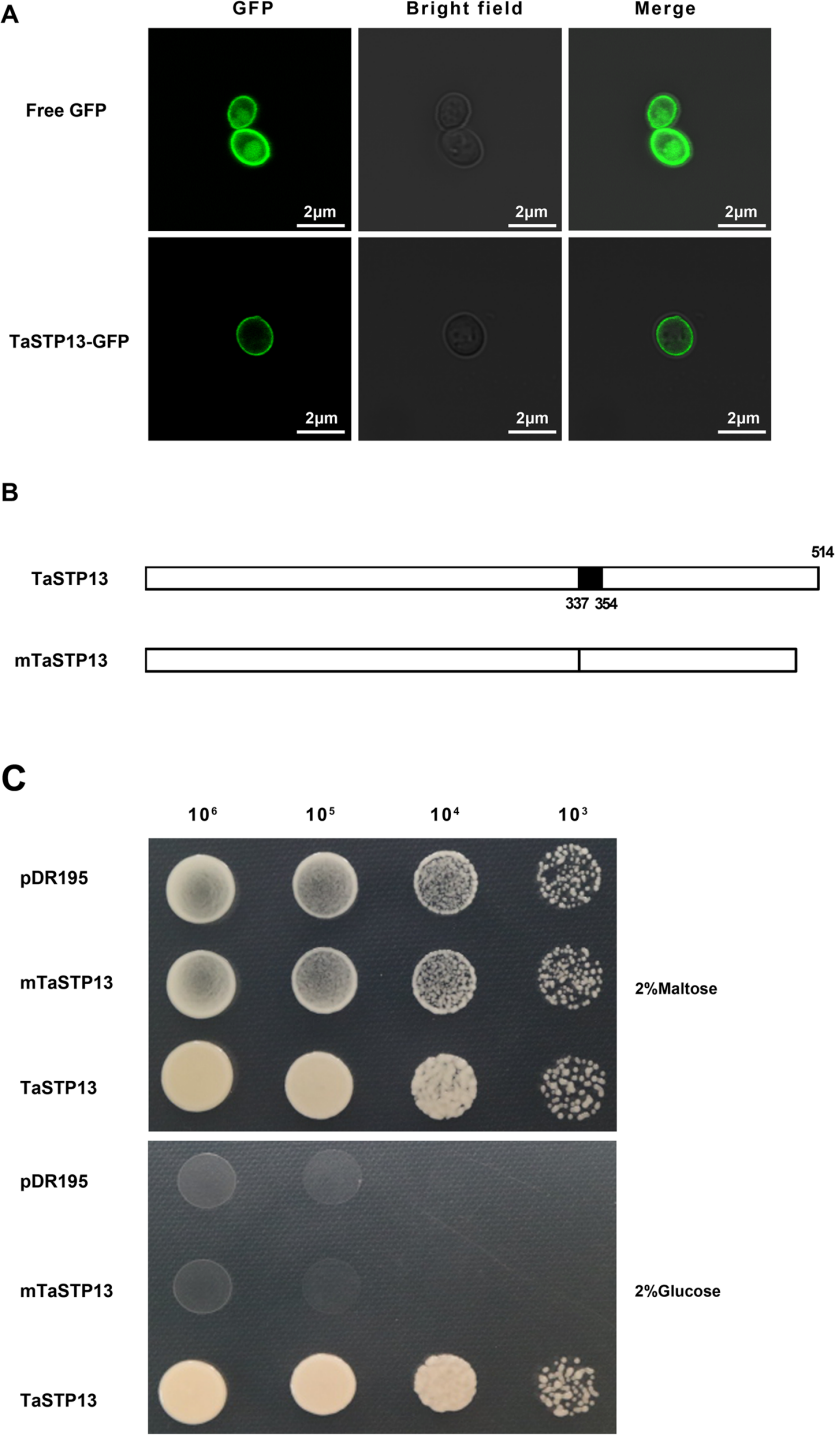
Expression of *TaSTP13* in *S. cerevisiae.* **a** Localization of GFP and TaSTP13-GFP in EBY.VW4000. Bright field and GFP fluorescence images merged and were taken by confocal microscopy. **b** Schematic diagrams of TaSTP13 deletions. The numbers of the amino acid residues in these truncated TaSTP13 proteins are indicated. mTaSTP13, (amino acid 337 to 354) deletion. **c** Growth of EBY.VW4000 carrying vector pDR195, plasmid pDR195::*mTaSTP13* and pDR195::*TaSTP13* on maltose (control), as well as 2% (w/v) of glucose. These experiments were repeated two times with similar results

To clarify whether it is appropriate to identify the function of TaSTP13 in yeast, the TaSTP13-GFP fusion construct was transformed into a yeast (*S. cerevisiae*) mutant, EBY.VW4000, which lacks hexose transporters and has no detectable hexose transport activity [18]. Fluorescence signals were monitored using confocal microscopy. Results revealed that TaSTP13-GFP is also localized in the plasma membrane when expressed in yeast (Fig. 3a), which is similar to what we found in plant cells. Thus, the transport properties of TaSTP13 may be determined in yeast.

The domain of TaSTP13 was analyzed using Interpro and ExPASy softwares. The results showed that a region of 18 amino acids (337–354) was possibly important for TaSTP13 (Fig. 3b). To assess whether this domain is pivotal for the transport activity of TaSTP13, *mTaSTP13* (*TaSTP13* lacking this region, Fig. 3b) was amplified using overlap-PCR and ligated into the pDR195 vector. The recombinant plasmid pDR195-*mTaSTP13* was introduced into the EBY.VW4000 mutant. The growth of positive transformants was measured on synthetic dropout (SD) containing glucose as the sole carbon source. The results showed that there was no colony formation observed in the yeast strains harboring pDR195-*mTaSTP13* or empty pDR195 plasmid (Fig. 3c), whereas the complemented strain carrying pDR195-*TaSTP13* could grow normally on media with glucose as the sole carbon source (Fig. 3c). These results indicate that this domain (amino acids 337–354) is functionally essential for TaSTP13.

### Knockdown of *TaSTP13* enhances wheat resistance to *Pst*

**Fig. 4:**
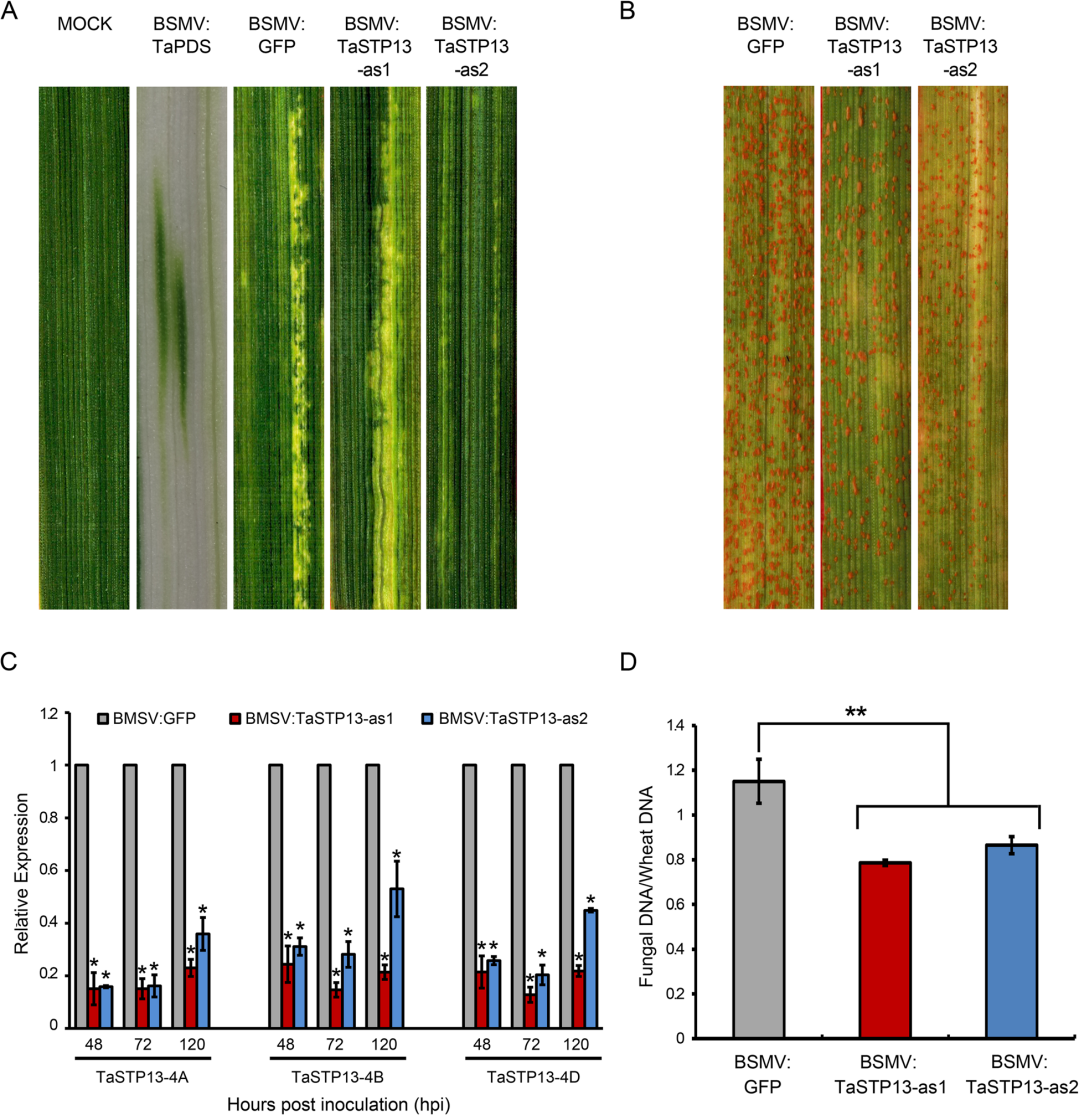
Silencing of *TaSTP13* in wheat–*Pst* interactions using the BSMV-VIGS system. **a** Mild chlorotic mosaic symptoms on BSMV-inoculated wheat leaves at 9–12 dpi; BSMV and photobleaching were evident in plants infected by BSMV:*TaPDS*. **b** Disease phenotypes of the fourth leaves pre-inoculated with BSMV:*GFP*, BSMV:*TaSTP13*-as1, and BSMV:*TaSTP13*-as2 at 14 dpi challenged with the *Pst* pathotype CYR31. **c** Relative transcript levels of *TaSTP13* in knock-down plants and control wheat leaves. RNA samples were isolated from the fourth leaves of wheat infected with BSMV:*GFP*, BSMV:*TaSTP13*-as1, and BSMV:*TaSTP13*-as2 at 48, 72 and 120 hpi with CYR31. The *TaEF1α* gene was used as an internal control. **d** Fungal and wheat biomass ratio measured via total DNA content at 14 dpi by absolute quantification using the internal reference *Pst* gene, *PstEF1*, and the wheat gene, *TaEF1α*. Values represent mean ± SD of three independent samples. Asterisks indicate a significant difference (*P < 0.05, **P < 0.01) from BSMV:*GFP*. Differences were assessed using Student’s *t*-test

In order to identify the function of *TaSTP13* in wheat leaves challenged with *Pst*, VIGS was utilized in this study. To make sure the three copies of *TaSTP13* were co-silenced, two *TaSTP13*-specific fragments were selected (Additional file 1: Figure S1). As shown in Fig. 4a, BSMV-inoculated wheat plants exhibited mild chlorotic mosaic symptoms, and there was an apparent photobleaching phenotype visualized in BSMV:*TaPDS*-infected plants at 12 days post-inoculation (dpi) (Fig. 4a), which was used as the control for VIGS efficiency. Subsequently, the surface of fourth leaves was infected with CYR31. Reduced rust phenotypes were observed in *TaSTP13*-silenced wheat seedlings at 14 dpi (Fig. 4b).

The qRT-PCR analysis revealed that the expression of the three copies of *TaSTP13* was significantly suppressed, and the silencing efficiency was roughly 50% higher (Fig. 4c). To examine whether the reduced disease symptom was involved in the mycelia development of wheat leaves, fungal biomass was measured by qRT-PCR. Results revealed that the *TaSTP13*-silenced wheat seedlings had lower fungal biomass compared to control plants (Fig. 4d). Altogether, these results indicate that down-regulation of *TaSTP13* leads to decreased wheat susceptibility to stripe rust in VIGS plants.

### Histology of fungal growth in *TaSTP13*-knockdown plants

**Fig. 5:**
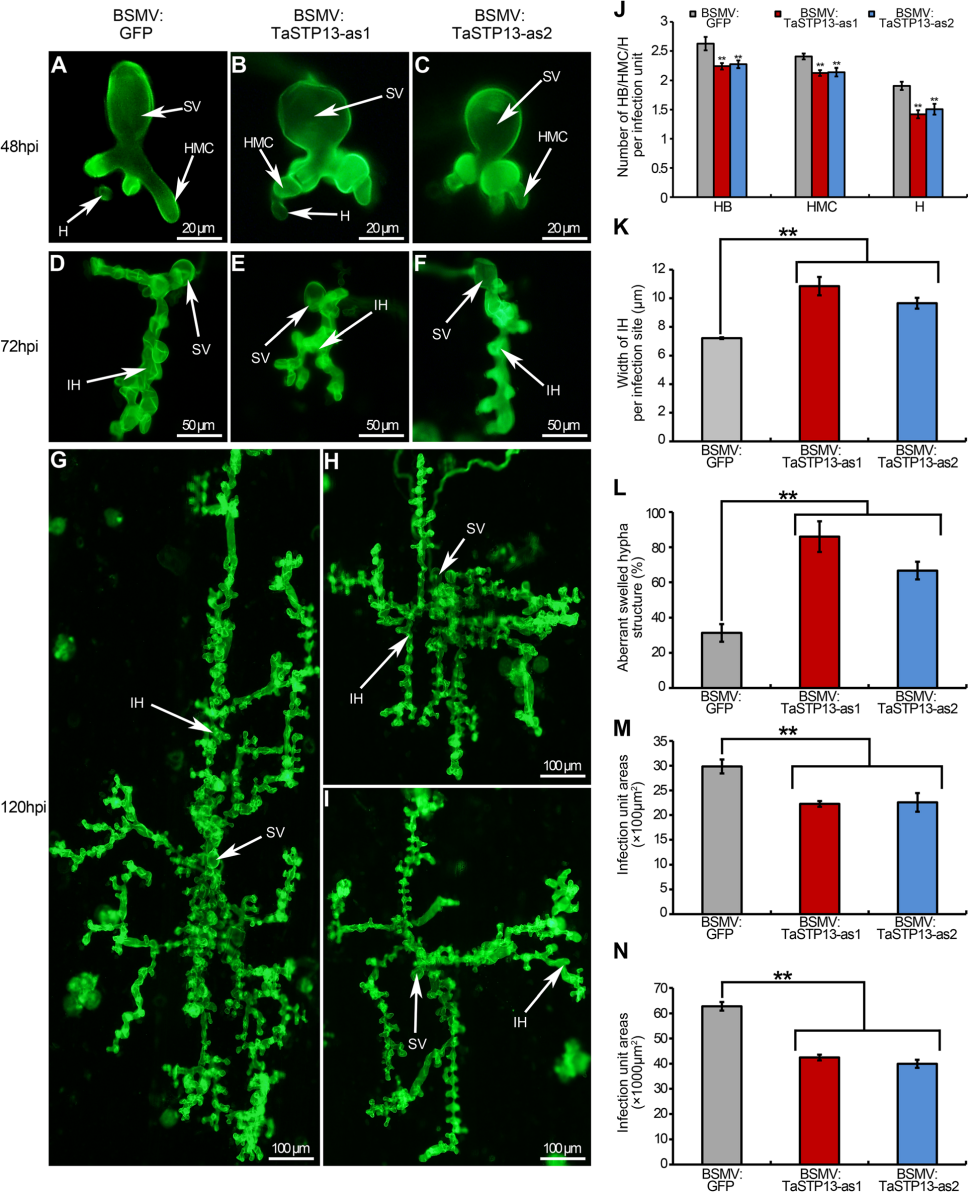
Histological observation of fungal growth and host response in wheat infected with BSMV:*GFP* and recombinant BSMV after inoculation with the *Pst* pathotype, CYR31. Growth of CYR31 in wheat leaves inoculated with BSMV:*GFP*, BSMV:*TaSTP13*-as1, and BSMV:*TaSTP13*-as2 at 48 hpi (**a–c**), 72 hpi (**d–f**), and 120 hpi (**g–i**) was observed under a fluorescence microscope. **j** The numbers of branches of infection hyphae (HB), haustorial mother cells (HMC) and haustoria (H) per infection unit were recorded at 48 hpi. **k** The width of infection hyphae (IH) in TaSTP13-silenced plants at 48 hpi with the *Pst* pathogtype CYR31. The width of IH was approximately perpendicular to substomatal vesicle (SV) and the apex of IH. **l** The ratio of aberrant swelled hypha structure in wheat inoculated with BMSV constructs at 48 hpi. The aberrant swelled hypha structure was defined as hypha, of which, width was larger than the mean width of IH in BSMV:*GFP*-infected wheat. *TaSTP13*-silenced plants had a significantly reduced infection unit area at 72 hpi (**m**) and 120 hpi (**n**). Values represent the mean ± SD of three independent samples with 50 infection sites each time. Asterisks indicate a significant difference (**P < 0.01) from BSMV: *GFP* inoculated plants. Differences were assessed using a one-way ANOVA and Student’s *t*-test

To clarify the reduction in the disease phenotype of *TaSTP13-*knockdown wheat plants infected with CYR31, fungal development at different *Pst* infection stages was microscopically detected. At 48 hpi, hyphal branches, haustorial mother cells, and haustorias were significantly decreased in *TaSTP13*-silenced plants (Fig. 5a-c, j). However, the hyphal length was similar to control plants at 48 hpi (Additional file 6: Figure S6). Additionally, fungal hypha was significantly wider and had aberrant swelling structures were observed in the *TaSTP13*-knockdown plants (Fig. 5a, c, k-l). At 72 and 120 hpi, hyphal spread*s* in *TaSTP13-*silenced seedlings were strictly limited compared with control plants (Fig. 5d-f, g-i, m-n). These results indicate that knockdown of *TaSTP13* triggered blocked hyphal development, leading to the weakened disease phenotype.

### Overexpression of *TaSTP13* promotes *Arabidopsis* susceptibility to powdery mildew

**Fig. 6:**
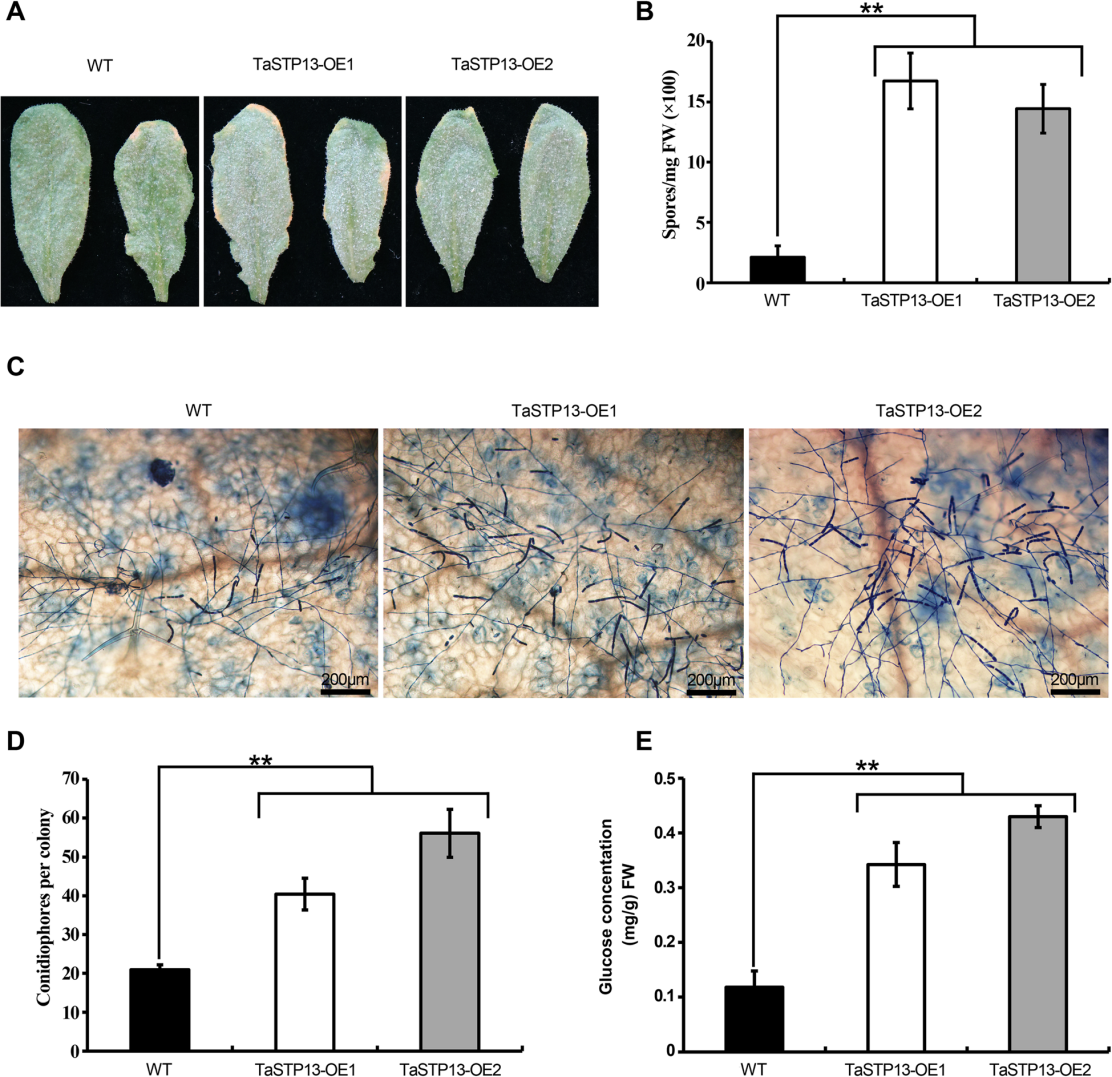
Overexpression of *TaSTP13* resulted in increased *Arabidopsis* susceptibility to powdery mildew. **a** Representative images of *Arabidopsis* leaves of indicated genotypes infected with tobacco powdery mildew at 12 dpi. Note, *TaSTP13* overexpression lines were more susceptible than Col-0. **b** Quantification of spore production in the indicated genotypes at 12 dpi normalized to leaf fresh weight (FW). Data represent the mean ± SD of three samples (*n* = 3, four leaves each) from one experiment, which was repeated three times with similar results. **c** Representative microscopic images of single colonies of powdery mildew on leaves of indicated genotypes at 5 dpi. Fungal structures were stained by trypan blue. Bars, 200 μm. **d** Total number of conidiophores per colony on leaves of indicated genotypes at 5 dpi. The bar-chart shows combined data from three independent experiments (at least 50 colonies were counted for each genotype per experiment). **e** Glucose concentrations in *TaSTP13* overexpression (OE) lines and wild-type leaves. Values are the mean ± SD of three independent biological replicates. Double asterisks indicate a significant difference (*P* < 0.01) according to the Student’s *t*-test and one-way ANOVA in comparison with WT.

To further investigate the possible function of *TaSTP13* in plant-fungal interactions, we generated several transgenic lines (TaSTP13-OE) overexpressing *TaSTP13* by introducing the *TaSTP13* overexpression construct into *Arabidopsis*. Expression of *TaSTP13* in different transgenic lines in the T3 generation was confirmed using RT-PCR (Additional file 7: Figure S7). Two transgenic lines were then inoculated with tobacco powdery mildew strain *Golovinomyces cichoracearum* (*Gc*) SICAU1 [19]. We found that the TaSTP13-OE plants showed clear enhanced disease susceptibility (Fig. 6a-b). Consistent with the results at 12 dpi, TaSTP13-OE plants had significantly more conidiophores per colony than Col-0 during the early infection stage at 5 dpi when the fungus began asexual reproduction (Fig. 6c-d). Taken together, overexpression of *TaSTP13* can promote *Arabidopsis* susceptibility to powdery mildew.

Since plasma membrane-localized TaSTP13 can transport hexoses into yeast cells, it is reasonable to speculate that up-regulation of *TaSTP13* induced by pathogens promotes hexose accumulation in plants. To test this hypothesis, sugar concentration of leaves from these transgenic lines was then analyzed by HPLC. The results showed that glucose content in *TaSTP13* overexpression plants was significantly increased compared with the wild-type control (Fig. 6e). Additionally, there was no obvious difference observed for other sugars (data not shown). These results indicate that TaSTP13 is a functional sugar transporter in plants.

### Oligomerization of TaSTP13

**Fig. 7:**
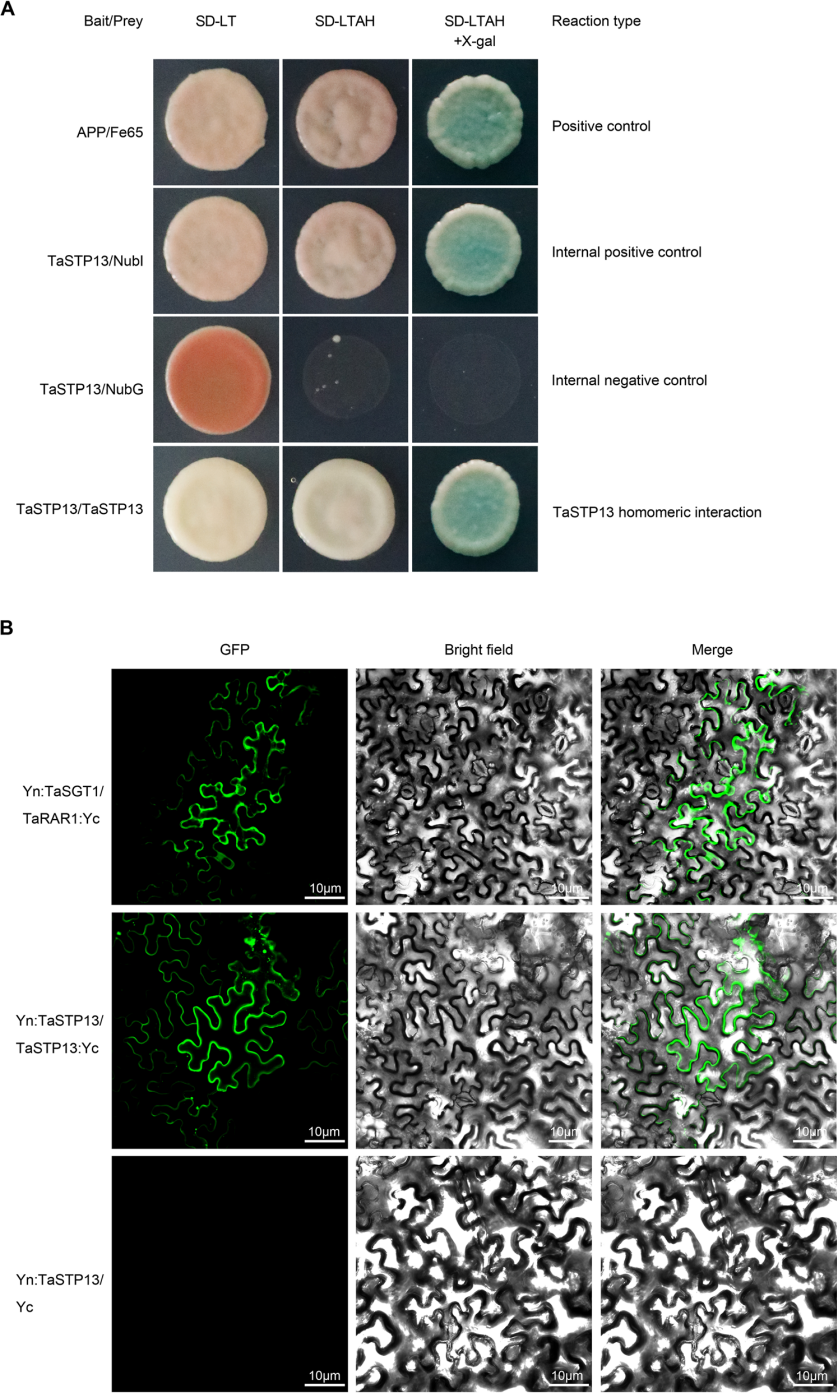
Homo-oligomerization of TaSTP13. **a** TaSTP13 can form homodimers within itself detected by the split-ubiquitin system using LacZ as a reporter gene. APP (amyloid A4 precursor protein) and Fe65 (amyloid beta A4 precursor protein-binding family B member 1) were used as positive control. Interactions of a *TaSTP13*-Cub fusion with a WT variant of NubI (internal positive control) or mutant variant of NubG (internal negative control) and *TaSTP13*-Nub fusion were tested. Cells of yeast strain NMY51 harboring the indicated plasmid combinations were grown on selective media (containing X–gal). Positive interaction was visualized by β-galactosidase expression in drop assays. Yeast growth assays on an SD medium (− Leu, − Trp, − Ade, and - His). **b** BiFC assays for TaSTP13 homooligomerization. Yn:*TaSGT1* + *TaRAR1*:Yc (positive control) is shown in the top three panels, Yn:*TaSTP13* + *TaSTP13*:Yc is shown in the middle three panels, and Yn:*TaSTP13* + Yc (negative control) is shown in the lower three panels. Agrobacterium-mediated transient expression of indicated constructs in *N. benthamiana* leaves. Bright field and YFP fluorescence (in green) images were taken by confocal microscopy and merged. All assays were repeated independently at least two times with comparable results

As reported, STPs can form dimers by themselves [15]. It is therefore conceivable that TaSTP13 functions as a dimer. Thus, TaSTP13 was fused with a mutated N-terminal half-ubiquitin protein (NubG) and C-terminal half-ubiquitin protein (Cub). The oligomerization of TaSTP13 was tested on SD media (−Trp, −Leu, −Ade, and -His) containing X-Gal by monitoring yeast cell growth. The cells co-transformed with *TaSTP13*-pPR3N (−NubG) and pBT3N-*TaSTP13* (−Cub) and could grow on the aforementioned media (Fig. 7a).

To evaluate the dimerization of TaSTP13 in plants, BiFC was carried out. TaSTP13 was fused with nYFP and cYFP sequences, and fusion proteins were transiently co-expressed in tobacco leaves. Strong green fluorescence signals were observed in the positive controls (TaSGT1 and TaRAR1) and in leaves co-expressing Yn:*TaSTP13* and *TaSTP13*:Yc (Fig. 7b). In contrast, there was no fluorescence visualized in the negative controls. Thus, the results corroborate our hypothesis that TaSTP13 proteins can form homo-oligomeric complexes.

In addition, to further determine whether TaSTP13 forms hetero-oligomers with other STPs from wheat, interactions between TaSTP13 and TaSTP6, a STP conferring enhanced wheat susceptibility to stripe rust [20], were studied in yeast and tobacco plants as described above. The results showed that TaSTP13 could not interact with TaSTP6 (data not shown), indicating that TaSTP13 possibly functions as a homo-oligomer.

## Discussion

Although the wheat *Lr67* gene (a natural mutation of *TaSTP13*) has been identified as an adult plant resistance gene to all three wheat rust pathogen species and powdery mildew [15], there has been no direct evidence that has characterized the functions of *TaSTP13* in wheat-rust fungi interactions. In the present study, *TaSTP13* was cloned, and its expression pattern was analyzed under a variety of treatments. In addition, the function of *TaSTP13* was investigated by a BSMV-VIGS system in *Pst*-infected wheat and heterologous overexpression in *Arabidopsis*. Our results suggest that *TaSTP13* may contribute to wheat susceptibility to stripe rust by increasing cytoplasmic hexose accumulation.

The previously reported STP family members were shown to be plasma membrane-localized monosaccharide/H^+^ symporters [7]. Results from this study revealed that TaSTP13 shares high sequence similarity with STP13-like proteins from other plants, and these proteins form a unique clade that is different from other STP family members, which exhibit highly conserved STP13 sequences throughout their evolution. Thus, it is inferred that *TaSTP13* potentially encodes an STP13-like proteins. Moore et al. found that TaSTP13 could accept glucose, fructose, mannose, and galactose as substrates [15]. This broad substrate specificity is similar to AtSTP13 from *Arabidopsis* [21] and HvSTP13 from barley [22]. Subcellular localization analysis in wheat protoplasts, tobacco epidermal cells, and yeast showed that TaSTP13 is localized in the plasma membrane, which is consistent with previously characterized STPs [1]. Altogether, these results indicate that TaSTP13 is a broad-spectrum monosaccharide transporter localized in the plasma membrane.

In order to remain active, membrane proteins need to assemble into polymers either transiently or permanently [23]. Oligomerization is thought to play an important role in the regulation of sugar transport properties and possibly contribute to protein stability [23]. Numerous studies have reported that plant sugar transporters generally exist as homo-oligomers [15]. In this study, TaSTP13 was demonstrated to be capable of forming oligomeric structures in BiFC and Y2H experiments, which is consistent with sugar transporters found in some STP and SWEET family members [15, 24]. Recently, LR67 has been reported to restrict wheat glucose uptake through heterodimerization with functional transporters (i.e., TaSTP13) [15, 22], indicating that the homo-oligomerization of TaSTP13 is indispensable for the import of hexose from the apoplast to the cytoplasm as mediated by TaSTP13.

Sugar transport and partitioning elicited by sugar transporters is one of the most important physiological processes for plant development and response to biotic and abiotic stress [1, 3, 4]. Numerous studies have found that STPs are regulated by pathogens and participate in sugar transport during plant-pathogen interactions [7]. For example, *AtSTP4* from *Arabidopsis* is transcriptionally induced after infection by *E. cichoracearum* and *Pseudomonas syringae* pv *tabaci* [11, 25]*.* Moreover, expression of *AtSTP13* is up-regulated by *P. syringae* pv. *tomato* DC3000 and *B. cinerea* [13, 21]. In grapevine, the transcription level of a hexose transporter gene, *VvHT5*, was significantly increased after powdery and downy mildew infection [12]. *TaSTP13* also had high expression levels in wheat during the early stages of leaf rust infection [26]. Similarly, results from this study revealed that the expression of *TaSTP13* was strongly induced in *Pst-*infected wheat leaves, suggesting that *TaSTP13* may be involved in sugar transport in wheat-rust interactions. In addition, the transcript abundance of *Lr67* was also increased in wheat leaves infected with leaf rust [15]. The transcriptional difference seems to indicate that *TaSTP13* responds differently to different rust infection.

Additionally, STPs also respond to abiotic factors, although their precise roles in regulating sugar transport during abiotic stress remain poorly understood at the molecular level [1]. In *Arabidopsis*, increasing *AtSTP3* and *AtSTP4* expression can transiently enhance response to wounding [25, 27]. *AtSTP1* and *AtSTP14* transcript levels displayed circadian oscillations and were found to be strongly dark-inducible [10, 28]. Additionally, the expression of *AtSTP13* has been found to be up-regulated by many abiotic factors, including multifarious elicitors, chemicals, hormones (i.e., ABA, MJ, GA biosynthesis inhibitor, PAC, and SA), and stress conditions (i.e., cold, heat, hypoxia, osmotic, oxidative, and salt) [1, 29]. The transcriptional responses of *VvHT5* were induced by wounding and ABA treatment [12]. Recently, it was reported that the transcript abundance of *HvSTP13* from barley was increased by salt and ABA [22]. In this study, *TaSTP13* was found to be strongly induced by wounding, LT, PEG, and certain exogenous hormone treatments, which was not concerned by Moore et al. [15]. Thus, it can be speculated that sugar compartmentation mediated by TaSTP13 may be associated with stress adaptation in unfavorable conditions.

Previous studies indicated that STP13-like proteins play an important role in sugar transport during plant-pathogen interactions [21, 22]. Whether STP13-like proteins contribute to host susceptibility or resistance appears to depend on the site where pathogens acquire sugar, although these STP13-like proteins have always been found to transport sugar from the apoplast to the cytoplasm. For example, increased AtSTP13 transport activity confers *Arabidopsis* enhanced resistance to *B. cinerea* and *P. syringae* pv. *tomato* DC3000 by reducing apoplastic hexoses [13, 21]. However, LR67 provides resistance to haustoria-forming pathogens, rust fungi, and powdery mildew by stealing sugar form host cytoplasms in wheat and decreasing the transport activity of functional transporters encoded by *Lr67* alleles [13, 21]. In the present study, the role of *TaSTP13* in wheat-*Pst* interactions was determined by a BSMV-VIGS system. The reduced disease symptoms, aberrant swelling structure in hyphae, and restricted colony size indicate that *TaSTP13* plays a pivotal role during *Pst* infection in wheat. It is reported that STPs (hexose/H^+^ symporters) can catalyze the uptake of hexose from the apoplast to the cytoplasm [1]. Therefore we may infer that cytoplasmic sugar concentration was increased in the *TaSTP13* overexpression *Arabidopsis* leaves. In addition, biotrophic fungi such as rust fungi or powdery mildew acquire nutrients via specialized feeding structures called haustorium by which biotrophic pathogens make intimate contact with the host cell membrane and allow for nutrient uptake [30, 31]. Therefore we infer that up-regulation of *TaSTP13* may promote *Arabidopsis* susceptibility to powdery mildew by increasing fungal sugar supply. Compared with the previous results reported by Moore et al. [15], these results further indicate that Lr67-mediated wheat rust resistance is possibly due to reduced sugar transport activity of TaSTP13. In addition, it should be pointed out that no obvious growth defects were observed in *TaSTP13*-silenced wheat plants, consistent with previous findings in *Arabidopsis* [21, 32]. One possible reason is physiologically functional redundancy among STPs. Interestingly, the functional redundancy of STPs seems not to be high in plant-pathogen interactions, possibly because contributions of different STP family members are distinct in different pathosystems.

## Conclusion

Together with the results of this study and previous reports provide enough circumstantial evidence to infer that *TaSTP13* plays a key role in wheat-*Pst* interactions and may be involved in the enhanced import of apoplastic hexoses into *Pst*-invaded cells. Thus, *TaSTP13* may serve as a candidate gene edited to create rust-resistant wheat cultivars using the CRISPR/Cas9 system. In addition, the underlying mechanisms require further investigation. The identification of TaSTP13 target proteins and specific transcription factors that regulate its expression should be a goal of future research in order to complete our understanding of the exact functional mechanism.

## Methods

### Plant materials, inoculation, and treatments

The *Arabidopsis thaliana* ecotype Columbia-0, *Nicotiana benthamiana*, and wheat cultivar Suwon 11 (Su11) were obtained from the Prof. Zhensheng Kang’s Lab (Northwest A&F University, China) and were used in this study. Tobacco powdery mildew isolate *Gc* SICAU1 was kindly provided by Prof. Wenming Wang’s Lab (Sichuan Agricultural University, China) and was maintained on tobacco leaves at 23 °C (16 h light, 8 h dark) in a growth room. *Arabidopsis* and *N. benthamiana* were grown following the methods described by [33].

For the tissue-specific gene expression tests, the wheat plants were grown in a growth chamber at 16 °C for 14 days and were transferred to another growth chamber at 4 °C for 30 days. Root, leaf, flower, stem, flag, and spikelet tissues were then sampled at 1/2 of complete flowering stage after about 40 days at 23 °C. The wheat plants were grown in a growth chamber, under 65% relative humidity and 200 μmol m^− 2^ s^− 1^ white light intensity for a 16-h light:8-h dark photoperiod.

Su11 and the *Pst* pathotype, CYR31 (virulent), were used in the wheat–*Pst* interactions study. To study *TaSTP1* expression levels in wheat leaves infected by *Pst*, leaf tissues were inoculated and sampled following the methods described [33]. Time points were selected as described by [34].

For the different abiotic stressors (i.e., wounding, LT, PEG, and NaCl), 14-day-old wheat seedlings were used as described [33]. For the chemical treatments, 14-day-old seedlings were sprayed with 10 mM SA, 1 mM MeJA, 1 mM ETH, or 1 mM ABA in 0.1% (v/v) ethanol. For the parallel mock control, wheat leaves were treated with 0.1% (v/v) ethanol. Wheat leaves were isolated at 0, 2, 6, 12, 24, and 48 hpt for RNA isolation.

For each experimental treatment, three independent biological replications were performed.

### RNA extraction and gene expression analysis

Total RNA from wheat was extracted using a Quick RNA isolation Kit (Huayueyang Biotechnology Co., Ltd., Beijing, China). About a 2 μg aliquot of total RNA was used to synthesize the first-strand cDNA with a RevertAid First Strand cDNA Synthesis Kit (Thermo Fisher Scientific, Waltham, MA, USA) with an oligo (dT)_18_ primer. Expression levels of target genes were normalized to *TaEF-1α* (GenBank accession No: Q03033). All qRT-PCR reactions were performed in a 25-μL reaction mixture containing an UltraSYBR Mixture (CWBIO Co., Ltd., Beijing, China), 10 pmol each of the forward and reverse gene-specific primers (Additional file 8: Table S1), and 2 μL of diluted cDNA (1:20) that was reverse transcribed. Gene expression was quantified using a CFX Connect RT-PCR Detection System (Bio-Rad, Hercules, California, USA). The qRT-PCR analysis represented data from three biological replicates with each group containing three technical repeats. These data were analyzed by the comparative 2^-△△Ct^ method [35]. Statistical significance was evaluated by Student’s *t*-test.

### Cloning of TaSTP13 and sequence analysis

Specific primers (Additional file 9: Table S2) from ATG to TGA were designed according to the ORFs of *TaSTP13* mRNA. *TaSTP13* was PCR-amplified from the *Pst*-infected Su11 cDNA sample using PrimeSTAR Max DNA Polymerase (TAKARA, Beijing). The PCR products were subcloned into pMD 19-T vector (Takara, Beijing, China) and sequenced. The obtained fragments were aligned with the *T. aestivum* cv. Chinese Spring (CS) genome using data from Ensembl Plants (http://plants.ensembl.org) and International Wheat Genome Sequencing Consortium (https://urgi.versailles.inra.fr/blast), and chromosomal locations were predicted.

Multiple sequence alignments were carried out using DNAMAN v6.0 (Lynnon Biosoft, USA). Polygenetic relationships were inferred using the neighbour-joining (NJ) method and bootstrap testing with 1000 replicates using MEGA v7.0 software [36]. The amino acid sequences of *TaSTP13* were analyzed in ExPASy (http://www.expasy.org) to identify their physicochemical properties. The expression of *TaSTP13* in different wheat tissues was analyzed in the WheatExp (https://wheat.pw.usda.gov/WheatExp/).

### Constructs, primers, and strains

DNA constructs were generated following standard molecular biology protocols or Gateway technology (Invitrogen, California, USA). More details of the DNA constructs are listed in Additional file 8: Table S1. All primers and strains are listed in Additional file 9: Table S2.

### Subcellular localization

The construct pK7FWG2-*TaSTP13* was transformed into *Agrobacterium tumefaciens* strain GV3101 using heat shock. The positive transformants verified by PCR were cultured in LB media with spectomycin (50 μg/ml) and rifampicin (50 μg/ml) at 28 °C in a shaking incubator at 220 rpm for 48 h. The bacteria were pelleted by centrifugation and resuspended in infiltration medium (10 mM MgCl_2_, 10 mM 2-(N-morpholino) ethanesulfonic acid MES, 200 μM acetosyringone, pH 5.7) in the dark for 3 h at room temperature (RT) before infiltration. The *A. tumefaciens* carrying construct pK7FWG2-*TaSTP13* was infiltrated into tobacco (*N. benthamiana*) leaves. Leaves were immersed in PBS consisting of 5 μM FM4–64 N-(3-triethylammoniumpropyl)-4-(6-(4-(diethylamino) phenyl) hexatrienyl pyridinium dibromide (Invitrogen, USA) for 10 min before visualization of the plasma membrane via staining. Wheat leaf protoplast isolation and transient expression of pTF486-*TaSTP13* constructs were performed as described previouly [37]. GFP fluorescence was monitored by an Olympus FV1000 confocal laser microscope (Olympus, Tokyo, Japan) with an excitation laser at 488 nm after transformations at 18 h (protoplasts) and 2 days (leaves). All assays were independently repeated three times with comparable results.

### Functional domain analysis of TaSTP13 in yeast

The domain of TaSTP13 was analyzed in Interpro (https://www.ebi.ac.uk/interpro/) ExPASy (https://prosite.expasy.org/). *mTaSTP13* was cloned by overlapping PCR with pairs of internal primers listed in Additional file 9: Table S2.

The empty vector pDR195 [38], as well as the constructed vectors (i.e., pDR195-*TaSTP13*, pDR195-*mTaSTP13*, pDR195-*GFP* and pDR195-*TaSTP13*-*GFP*), were transformed into the hexose transport deficient *S. cerevisiae* mutant EBY.VW4000 [18]; these transformations were conducted using the LiAc method [39] for complementation and subcellular localization assays. Transformants were selected on SD media lacking uracil (6.7 g L^− 1^ yeast nitrogen base, 2 g L^− 1^ amino acid (uracil) drop out mix, 2% (w/v) maltose, and 1% (w/v) agar). Potential clones were further verified by PCR. To determine subcellular localization in *S. cerevisiae*, yeast cells harboring pDR195-*TaSTP13*-*GFP* and pDR195-*GFP* (control) were visualized with a laser scanning confocal microscope, as described above. Additionally, to examine whether the predicted domain was important for the transport activity of TaSTP13, positive transformants were grown on liquid SD medium supplemented with 2% (w/v) maltose for 1 day, and serial dilutions (i.e., 10^3^, 10^4^, 10^5^, and 10^6^ cells mL^− 1^ quantified with hemacytometer) were dropped on solid media containing glucose as the sole carbon source for 3 days at 30 °C.

### BSMV-mediated TaSTP13 gene silencing

Two specific VIGS sequence regions from the coding sequence (260 bp, nucleotides 34–293; 219 bp, nucleotides 1264–1482, Additional file 1: Figure S1) that showed the lowest sequence similarity with other wheat genes and the highest polymorphism within STP family in a BLASTN search of the NCBI were chosen to generate their γRNA-based derivative plasmids as described in Additional file 8: Table S1. The BSMV:*GFP* vector was used as the control. Viral RNA molecules were prepared as described [40]. Plants were infected with BSMV RNA (i.e., BSMV:*GFP*, BSMV:*TaPDS,* BSMV:*TaSTP13*-as1, and BSMV:*TaSTP13*-as2) following a modified protocol [41, 42]. At 10–12 days after virus inoculation, the fourth leaves were further treated with CYR31. Plants were then maintained at 16 °C, and fourth leaves were sampled at 48, 72, and 120 h post-inoculation (hpi) for histological observation and silencing efficiency. Gene-silencing efficiency analysis was conducted by qRT-PCR. At 14 days after pathogen inoculation, when extensive fungal growth was visible on the leaves, infection phenotypes of *Pst* were photographed and collected for further analysis. Three independent inoculations were performed using 50 seedlings inoculated with each BSMV virus. Genomic DNA was extracted by the CTAB method [43]. Total genomic DNA was extracted from *Pst*-inoculated wheat leaves. The relative biomass ratio was acquired by comparing the *PsEF1* [44] copy numbers with *TaEF-1α* copy numbers.

All primers are listed in Additional file 9: Table S2.

### Histological observation of fungal growth

Wheat leaf segments were fixed and bleached as described previously [33]. Wheat germ agglutinin (WGA) conjugated to Alexa Fluor 488 (Invitrogen, Carlsbad, CA, USA) was used to stain the *Pst* infection structures as previously described [45]. The infection sites in stained tissues were examined using an Olympus BX-51 microscope (Olympus, Tokyo, Japan) and measured with the cellSens Entry software (Olympus, Tokyo, Japan). Data of each index were obtained as described previously [20]. For the statistical analyses, standard deviations, one-way ANOVA and *t*-test were performed using SPSS software v17.0 based on three independent samples.

### *Arabidopsis* transformation and inoculation

The pK7FWG2-*TaSTP13* construct was transferred into the *A. tumefaciens* strain, GV3101, by heat shock for subsequent floral dipping transformation in *Arabidopsis* [46]. Primary transformants (T1) were selected on ½MS medium containing 50 μg/ml kanamycin. Selection marker resistant seedlings were verified by microscope and RT-PCR. Seedlings were then transferred into single pots filled with soil and allowed to grow until the next generation of seeds was produced in a growth chamber. Two representative homozygous lines were further analyzed. Methods of inoculation with powdery mildew and conidiophore counting were the same as those described previously [47].

### Extraction and determination of water-soluble carbohydrates

The leaves of *Arabidopsis* for determination of water-soluble carbohydrates were collected as described previously [20]. Water-soluble carbohydrates were extracted, and high-performance liquid chromatography was performed as described [48].

### Split-ubiquitin analysis

Polymerization of TaSTP13 was tested by the split-ubiquitin Y2H system [49]. To ensure correct expression and functionality of this system, the “bait” construct, pBT3-N-*TaSTP13*, was co-transformed with the prey control vector, which expresses the wild-type NubI (internal positive control) portion and the NubG (internal negative control) portion. Self-interaction of TaSTP13 was determined by co-transformation of pBT3-N-*TaSTP13* and the “prey” vector, pPR3N-NubG-*TaSTP13.* The yeast colonies were grown at 30 °C on SD-Leu-Trp medium (SD-LT). Self-interaction of TaSTP13 was assessed by monitoring cell growth on SD-Leu-Trp-Ade-His medium (SD-LTAH) and SD-LTAH containing X-Gal for 4 days. Positive controls were used as described previously [20].

### BiFC assay

The ORF of *TaSTP13* was cloned into vectors pSPYNE(R)173 and pSPYCE(M) [50], respectively. Each pair of constructs was co-introduced into *N. benthamiana* leaves as described previously [24]. Two days after infiltration, YFP fluorescence was observed using a confocal laser scanning microscope (Olympus FV1000). Three independent biological repeats were carried out.

### Statistical analyses

Data analysis was completed using SPSS software v17.0. Statistical significance was determined by Student’s *t*-test for comparisons between two groups and one-way ANOVA (followed by LSD and Bonferroni test) for experiments with multiple treatments. In all figures, the spread of values is shown as error bars representing standard deviation of the means.

### Accession numbers

Sequence data used in this study can be found in the NCBI database (http://www.ncbi.nlm.nih.gov/) with the following accession numbers: *TaEF-1α* (Q03033), AtUBC21 (AT5G25760), TaSTP13-4A (ALL26328.1), TaSTP13-4B (ALL26329.1), TaSTP13-4D (All26330.1), SbSTP13 (XP_002465636.1), ZmSTP13 (NP_001310214.1), SiMST4 (XP_004985175.1), PhMST4 (XP_025795007.1), OsMST4 (XP_015630449.1), BdMST4 (XP_003558480.1), HvSTP13 (IPK Barlex accession: HORVU4Hr1G067450), VvHT5 (AAT09979.1), GmSTP13 (XP_003539275.1), NtSTP13 (XP_016500201.1), LeHT2 (CAB52689.1), StSTP13 (XP_006359910.1), AtSTP13 (NP_198006.1), AtSTP1 (NP_172592.1), AtSTP2 (NP_172214.5), AtSTP3 (NP_200960.2), AtSTP4 (NP_188627.1), AtSTP5 (NP_174718.1), AtSTP6 (NP_187247.1), AtSTP7 (NP_192114.1), AtSTP8 (NP_197997.1), AtSTP9 (NP_175449.1), AtSTP10 (NP_188628.1), AtSTP11 (NP_197718.1), AtSTP12 (NP_193879.4), and AtSTP14 (NP_177845.1).

## Supplementary information

**Additional file 1: Figure S1.** Multi-alignment of the encoding sequences of three *TaSTP13* copies and two specific VIGS sequence regions. *TaSTP13-4A*, *TaSTP13-4B*, and *TaSTP13-4D* represent *TaSTP13* coding regions from wheat genomes A, B, and D, respectively. Identical and similar nucleotides are shaded in black and light gray, respectively. VIGS sites are indicated by a single line.

**Additional file 2: Figure S2.** Multi-alignment of the TaSTP13 proteins. TaSTP13-4A, TaSTP13-4B, and TaSTP13-4D represent deduced TaSTP13 proteins from the wheat genome A, B, and D, respectively. Identical and similar amino acid residues are shaded in black, light gray and pink, respectively.

**Additional file 3: Figure S3.** Multi-alignment of the TaSTP13 and LR67 protein. TaSTP13-4A, TaSTP13-4B, and TaSTP13-4D represent TaSTP13 proteins isolated from wheat genomes A, B, and D, respectively. Identical and similar nucleotide residues are shaded in black and light gray, respectively. The two amino acid residues that distinguish LR67 and TaSTP13 are blocked in a red frame.

**Additional file 4: Figure S4.** Phylogenetic analysis of TaSTP13. The phylogenetic tree of TaSTP13 was carried out with the MEGA7 by neighbour-joining approach. The confidence level for the groupings was estimated using 1000 bootstrap replicates. Branches are labeled with the protein names and GenBank accession numbers.

**Additional file 5: Figure S5.** Transcript profile of *TaSTP13-4B* in response to abiotic stress (A) and exogenous hormones (B). Transcript profile of *TaSTP13-4D* in response to abiotic stress (C) and exogenous hormones (D). Wheat leaves were sampled at 0, 2, 6, 12, 24 and 48 hpt. Expression levels were normalized to *TaEF-1a*. The relative expression of *TaSTP13* was calculated using the comparative threshold method (2^–ΔΔ*C*^_T_). Significant differences are indicated with asterisks (*P* < 0.01) according to Student’s *t*-test. Bars indicate the mean ± SD of three independent biological replicates. ABA, abscisic acid; SA, salicylic acid; ETH, ethylene; MeJA, methyl jasmonate; LT, low tempreture; PEG; polyethyleneglycol 6000.

**Additional file 6: Figure S6.** The length of IH in *TaSTP13*-silenced and control plants at 48 hpi. No significant difference in the length of IH was observed between control and *TaSTP13*-silenced plants. The length of IH was measured from the substomatal vesicle to the apex of the longest infection hyphae. Values are represented as the mean ± SD of three independent samples with 50 infection sites each. Significance was determined using one-way ANOVA.

**Additional file 7: Figure S7.** RT-PCR analysis of *TaSTP13* expression in TaSTP13-OE and wild type plants. *AtUBC21* was used as the control (bottom panel).

**Additional file 8: Table S1.** DNA constructs in this study.

**Additional file 9: Table S2.** The primers and strains used in this study.

## Data Availability

All data generated in this study are included in the paper and in the supporting information files.

## Abbreviations

ABA: Abscisic acid
BAK1: BRASSINOSTEROID INSENSITIVE 1-associated receptor kinase 1
BiFC: Bimolecular fluorescence complementation
CaMV: Cauliflower mosaic virus
CS: Chinese Spring
Cub: C-terminal half-ubiquitin protein
Dpi: Days post-inoculation
ETH: Ethylene MeJA Methyl jasmonate
FM4–64: N-(3-triethylammoniumpropyl)-4-(6-(4-(diethylamino) phenyl) hexatrienyl pyridinium dibromide
Hpi: Hours post-inoculation
Hpt: Hours post treatment
LSD: Least significant difference
LT: Low temperature
NJ: Neighbour-joining
NubG: N-terminal half-ubiquitin protein
ORF: Open reading frame
PEG: Polyethyleneglycol
*Pst*: *Puccinia striiformis* f. sp. *tritici*;
qRT-PCR: Quantitative real-time PCR
RT-PCR: Reverse transcription polymerase chain reaction
SA: Salicylic acid
SD: Synthetic dropout
STPs: Sugar transport proteins
VIGS: Virus-induced gene silencing
WGA: Wheat germ agglutinin
Y2H: Yeast two-hybrid
