# Supplementary material for: TaSTP13 contributes to wheat susceptibility to stripe rust possibly by increasing cytoplasmic hexose concentration
Source: BMC Plant Biol. 2020 Jan 30;20:49. doi: 10.1186/s12870-020-2248-2 (PMC6993525; doi:10.1186/s12870-020-2248-2)
Supplement: Supplementary file 1 — Additional file 1: Figure S1. Multi-alignment of the encoding sequences of three TaSTP13 copies and two specific VIGS sequence regions. TaSTP13-4A, TaSTP13-4B, and TaSTP13-4D represent TaSTP13 coding regions from wheat genomes A, B, and D, respectively. Identical and similar nucleotides are shaded in black and light gray, respectively. VIGS sites are indicated by a single line. [file 12870_2020_2248_MOESM1_ESM.docx]

**Supplementary information**

**
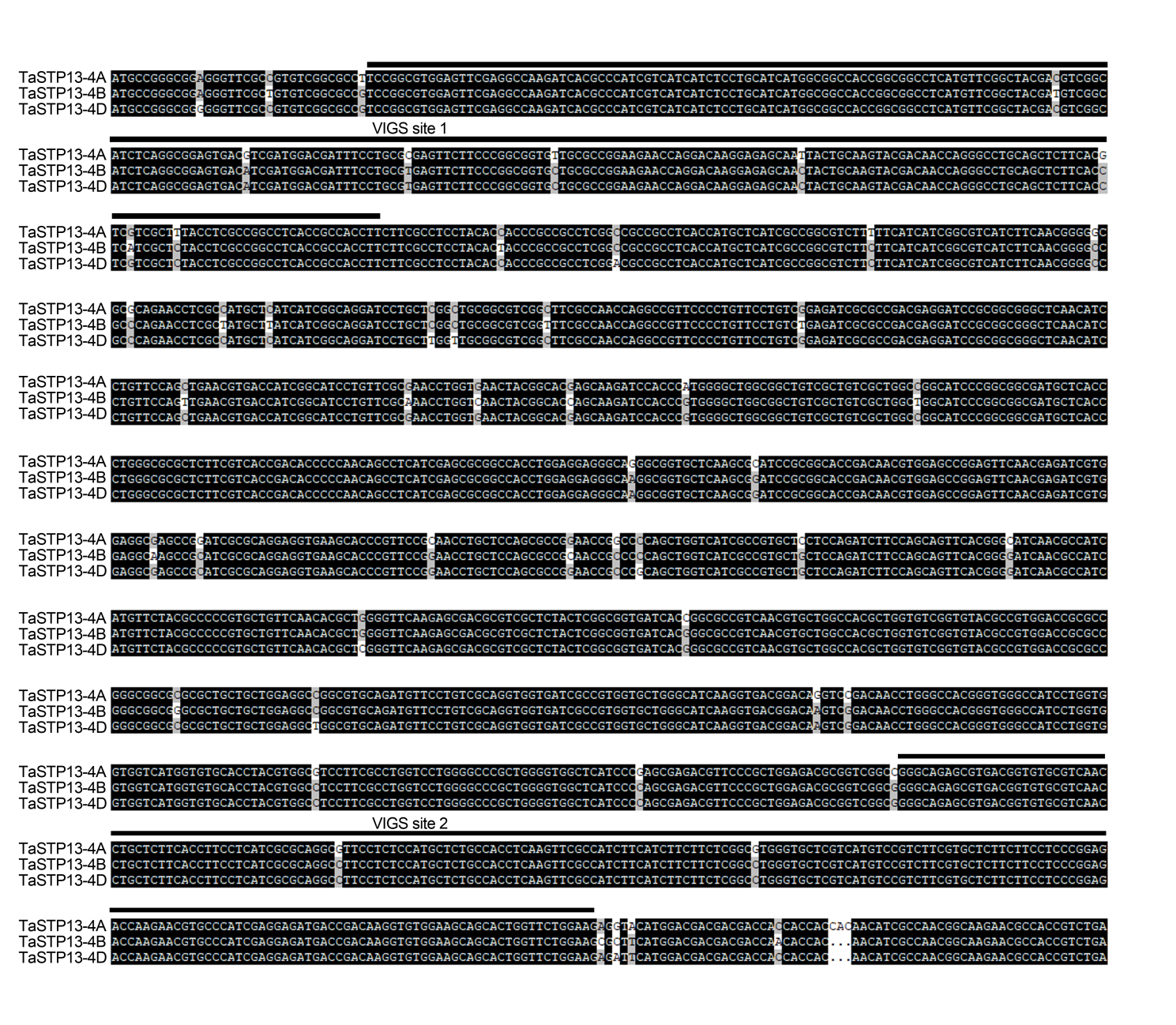
**

**Figure S1. Multi-alignment of the encoding sequences of three *TaSTP13* copies and two specific VIGS sequence regions.** *TaSTP13-4A*, *TaSTP13-4B*, and *TaSTP13-4D* represent *TaSTP13* coding regions from wheat genomes A, B, and D, respectively. Identical and similar nucleotides are shaded in black and light gray, respectively. VIGS sites are indicated by a single line.
