# Supplementary material for: TaSTP13 contributes to wheat susceptibility to stripe rust possibly by increasing cytoplasmic hexose concentration
Source: BMC Plant Biol. 2020 Jan 30;20:49. doi: 10.1186/s12870-020-2248-2 (PMC6993525; doi:10.1186/s12870-020-2248-2)
Supplement: Supplementary file 2 — Additional file 2: Figure S2. Multi-alignment of the TaSTP13 proteins. TaSTP13-4A, TaSTP13-4B, and TaSTP13-4D represent deduced TaSTP13 proteins from the wheat genome A, B, and D, respectively. Identical and similar amino acid residues are shaded in black, light gray and pink, respectively. [file 12870_2020_2248_MOESM2_ESM.docx]

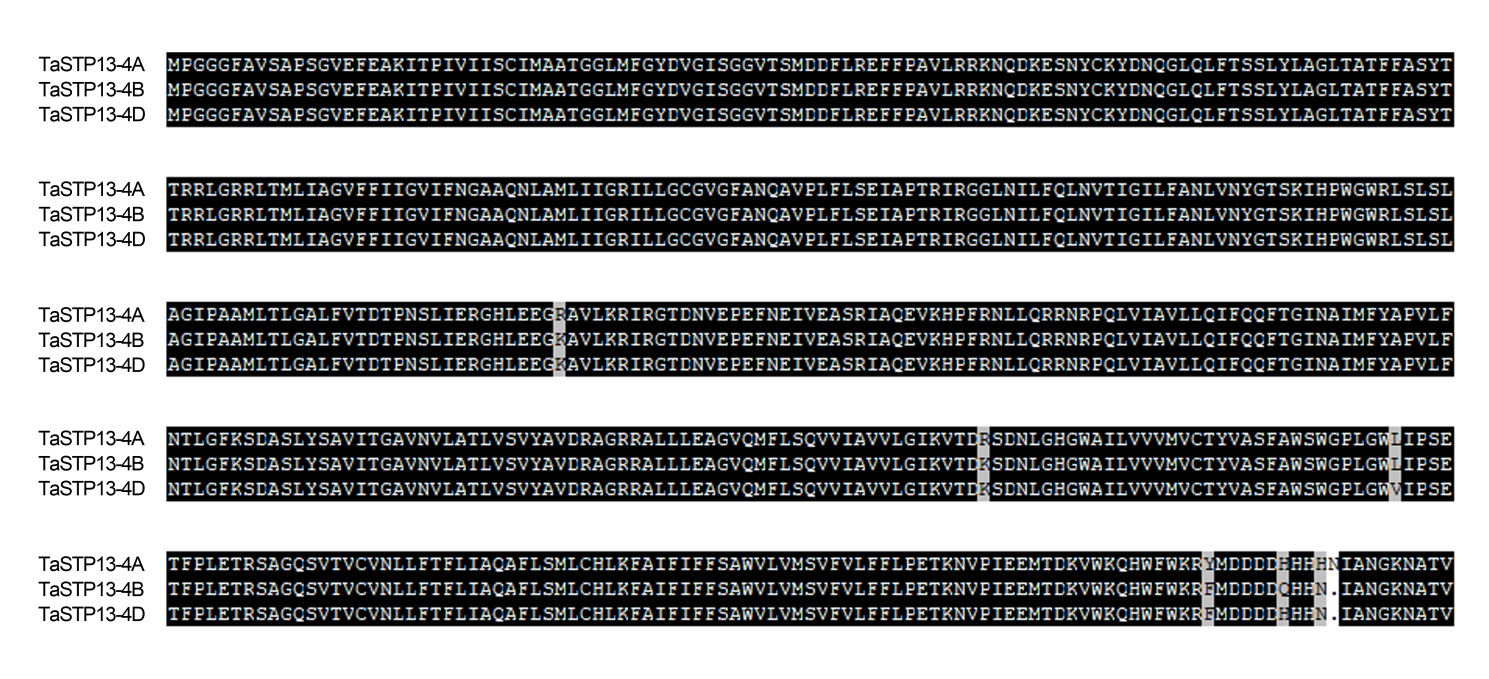


**Figure S2.** **Multi-alignment of the TaSTP13 proteins.** TaSTP13-4A, TaSTP13-4B, and TaSTP13-4D represent deduced TaSTP13 proteins from the wheat genome A, B, and D, respectively. Identical and similar amino acid residues are shaded in black, light gray and pink, respectively.
