# Supplementary material for: TaSTP13 contributes to wheat susceptibility to stripe rust possibly by increasing cytoplasmic hexose concentration
Source: BMC Plant Biol. 2020 Jan 30;20:49. doi: 10.1186/s12870-020-2248-2 (PMC6993525; doi:10.1186/s12870-020-2248-2)
Supplement: Supplementary file 3 — Additional file 3: Figure S3. Multi-alignment of the TaSTP13 and LR67 protein. TaSTP13-4A, TaSTP13-4B, and TaSTP13-4D represent TaSTP13 proteins isolated from wheat genomes A, B, and D, respectively. Identical and similar nucleotide residues are shaded in black and light gray, respectively. The two amino acid residues that distinguish LR67 and TaSTP13 are blocked in a red frame. [file 12870_2020_2248_MOESM3_ESM.docx]

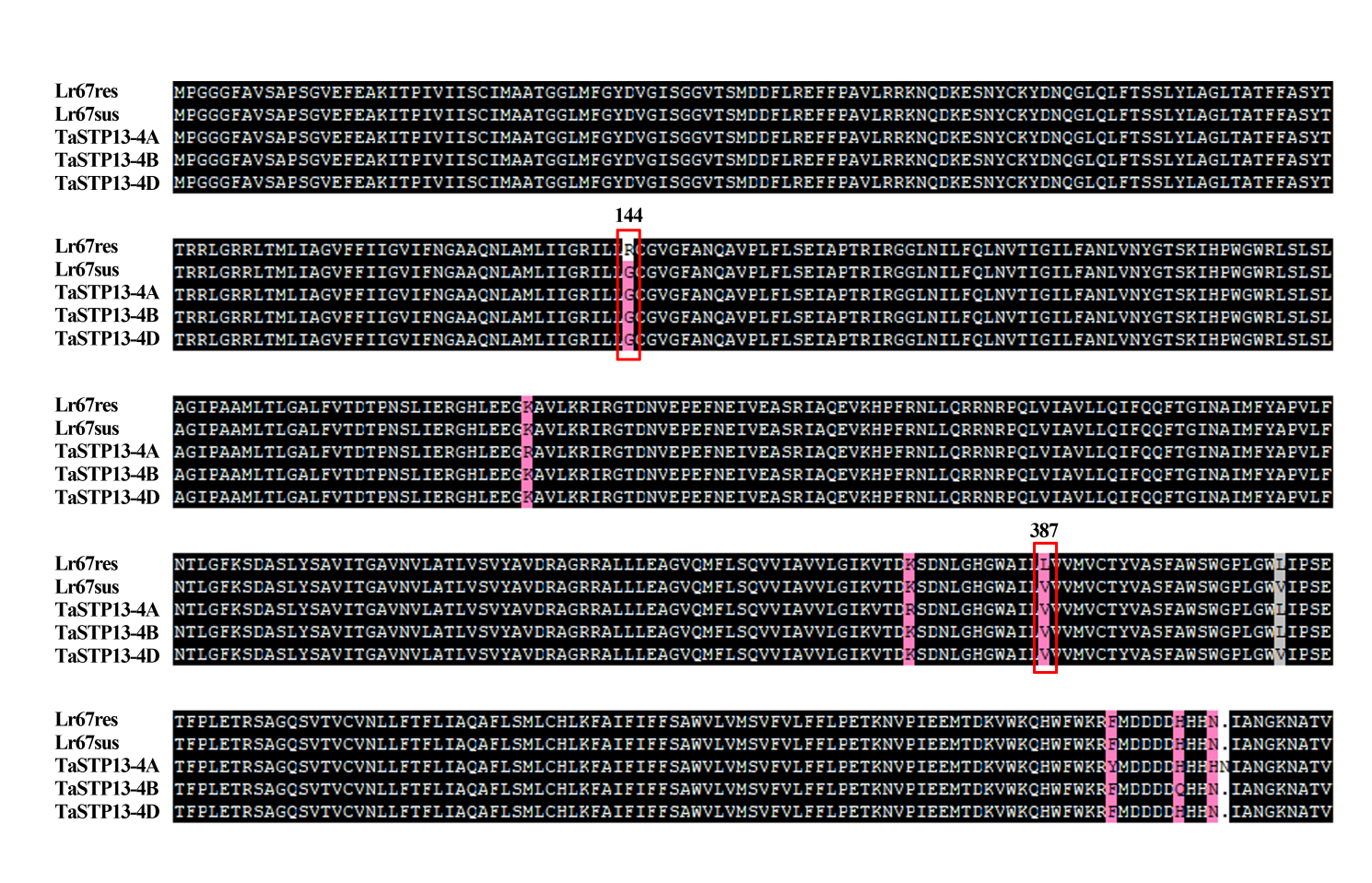


**Additional file 3 Figure S3. Multi-alignment of the TaSTP13 and LR67 protein.** TaSTP13-4A, TaSTP13-4B, and TaSTP13-4D represent TaSTP13 proteins isolated from wheat genomes A, B, and D, respectively. Identical and similar nucleotide residues are shaded in black and light gray, respectively. The two amino acid residues that distinguish LR67 and TaSTP13 are blocked in a red frame.
