# Supplementary material for: TaSTP13 contributes to wheat susceptibility to stripe rust possibly by increasing cytoplasmic hexose concentration
Source: BMC Plant Biol. 2020 Jan 30;20:49. doi: 10.1186/s12870-020-2248-2 (PMC6993525; doi:10.1186/s12870-020-2248-2)
Supplement: Supplementary file 4 — Additional file 4: Figure S4. Phylogenetic analysis of TaSTP13. The phylogenetic tree of TaSTP13 was carried out with the MEGA7 by neighbour-joining approach. The confidence level for the groupings was estimated using 1000 bootstrap replicates. Branches are labeled with the protein names and GenBank accession numbers. [file 12870_2020_2248_MOESM4_ESM.docx]

**
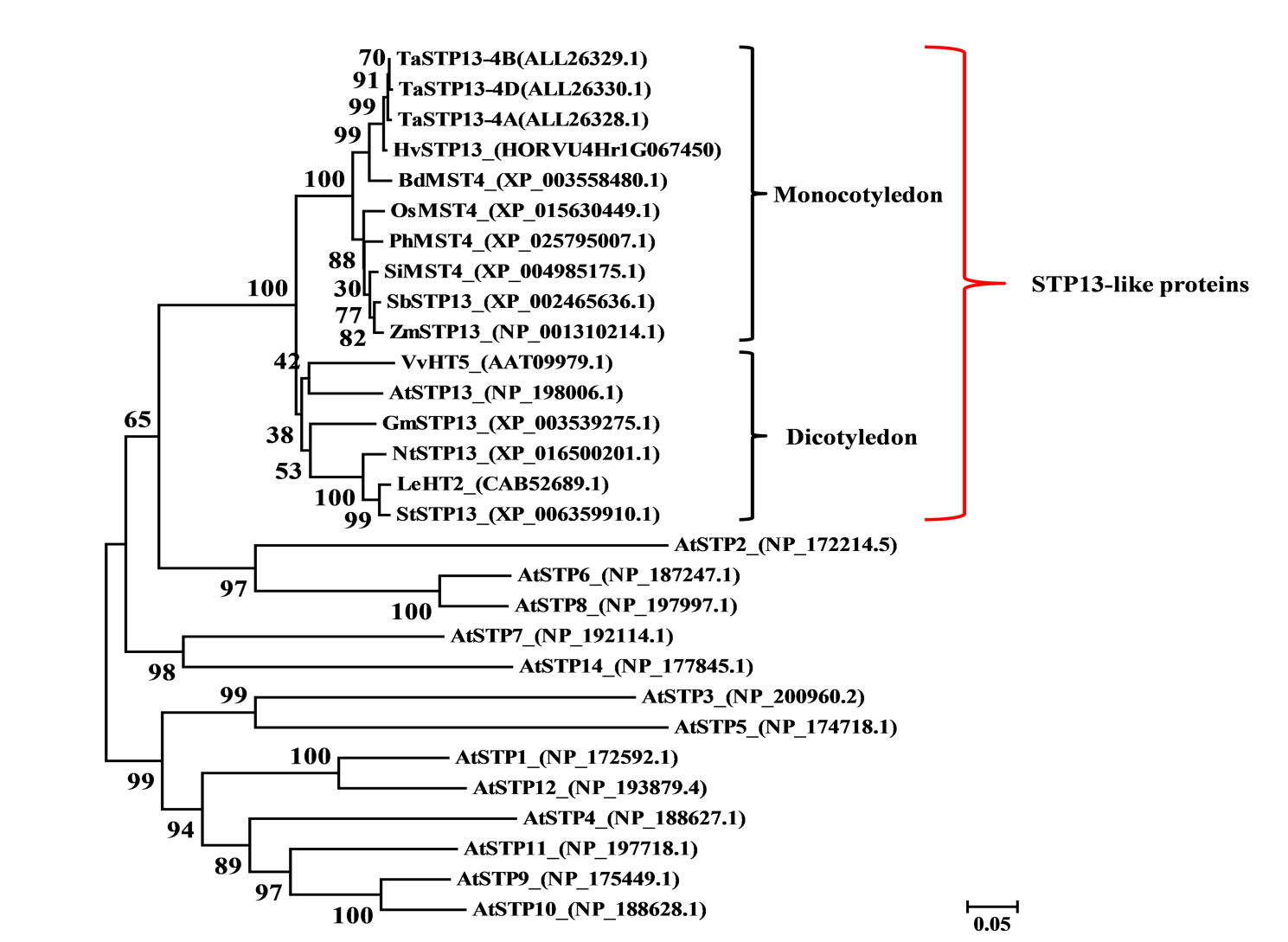
**

**Additional file 4. Figure S4. Phylogenetic analysis of TaSTP13.** The phylogenetic tree of TaSTP13 was carried out with the MEGA7 by neighbour-joining approach. The confidence level for the groupings was estimated using 1,000 bootstrap replicates. Branches are labeled with the protein names and GenBank accession numbers.
