# Supplementary material for: TaSTP13 contributes to wheat susceptibility to stripe rust possibly by increasing cytoplasmic hexose concentration
Source: BMC Plant Biol. 2020 Jan 30;20:49. doi: 10.1186/s12870-020-2248-2 (PMC6993525; doi:10.1186/s12870-020-2248-2)
Supplement: Supplementary file 5 — Additional file 5: Figure S5. Transcript profile of TaSTP13-4B in response to abiotic stress (A) and exogenous hormones (B). Transcript profile of TaSTP13-4D in response to abiotic stress (C) and exogenous hormones (D). Wheat leaves were sampled at 0, 2, 6, 12, 24 and 48 hpt. Expression levels were normalized to TaEF-1a. The relative expression of TaSTP13 was calculated using the comparative threshold method (2–ΔΔCT). Significant differences are indicated with asterisks (P < 0.01) according to Student’s t-test. Bars indicate the mean ± SD of three independent biological replicates. ABA, abscisic acid; SA, salicylic acid; ETH, ethylene; MeJA, methyl jasmonate; LT, low tempreture; PEG; polyethyleneglycol 6000. [file 12870_2020_2248_MOESM5_ESM.docx]

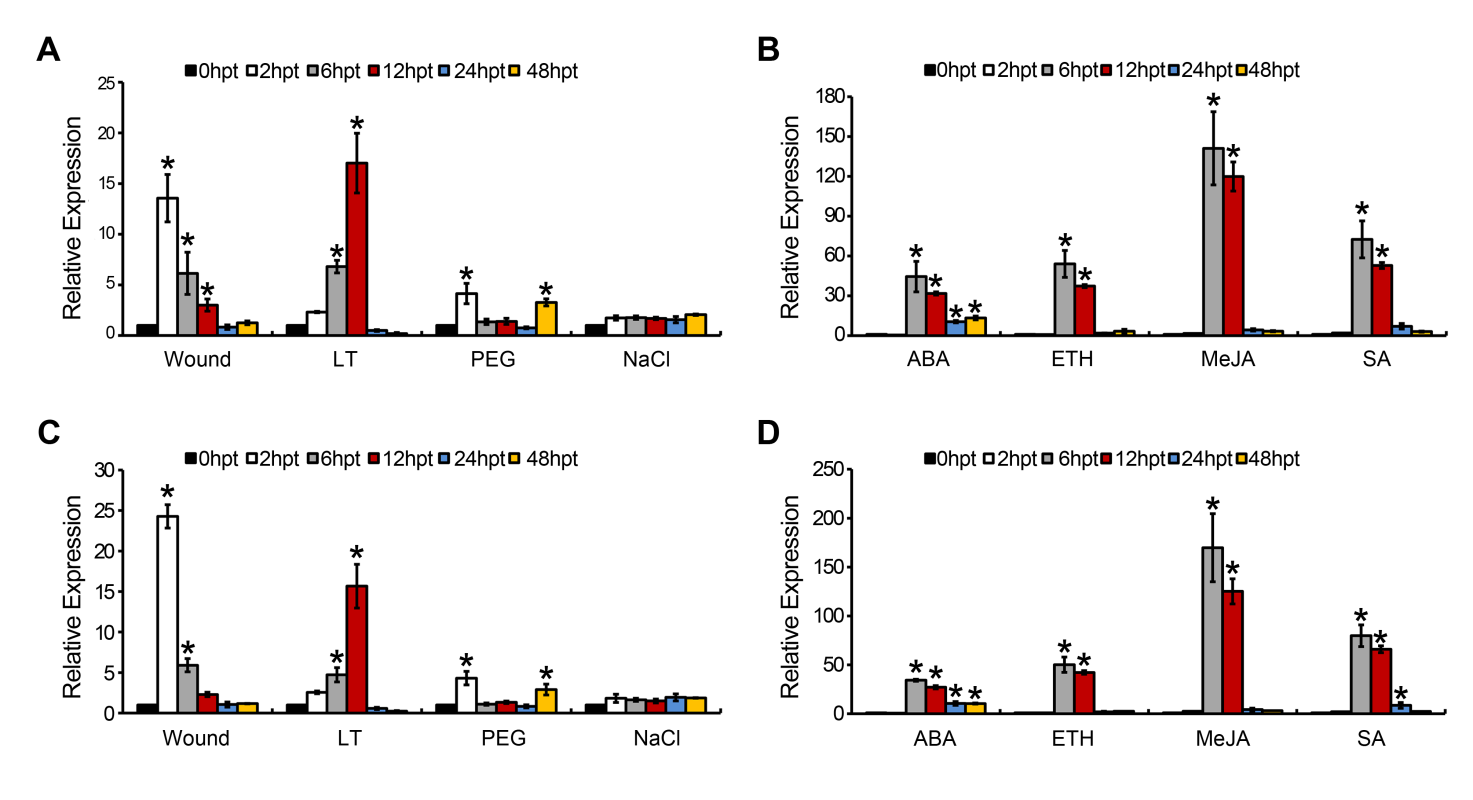


**Additional file 5. Figure S5. Transcript profile of *TaSTP13-4B* in response to abiotic stress (A) and exogenous hormones (B).** **Transcript profile of *TaSTP13-4D* in response to abiotic stress (C) and exogenous hormones (D).**Wheat leaves were sampled at 0, 2, 6, 12, 24 and 48 hpt. Expression levels were normalized to *TaEF-1a*. The relative expression of *TaSTP13* was calculated using the comparative threshold method (2^–ΔΔ^*^C^*_T_). Significant differences are indicated with asterisks (*P* < 0.01) according to Student’s *t*-test. Bars indicate the mean ± SD of three independent biological replicates. ABA, abscisic acid; SA, salicylic acid; ETH, ethylene; MeJA, methyl jasmonate; LT, low tempreture; PEG; polyethyleneglycol 6000.
