# Supplementary material for: TaSTP13 contributes to wheat susceptibility to stripe rust possibly by increasing cytoplasmic hexose concentration
Source: BMC Plant Biol. 2020 Jan 30;20:49. doi: 10.1186/s12870-020-2248-2 (PMC6993525; doi:10.1186/s12870-020-2248-2)
Supplement: Supplementary file 6 — Additional file 6: Figure S6. The length of IH in TaSTP13-silenced and control plants at 48 hpi. No significant difference in the length of IH was observed between control and TaSTP13-silenced plants. The length of IH was measured from the substomatal vesicle to the apex of the longest infection hyphae. Values are represented as the mean ± SD of three independent samples with 50 infection sites each. Significance was determined using one-way ANOVA. [file 12870_2020_2248_MOESM6_ESM.docx]

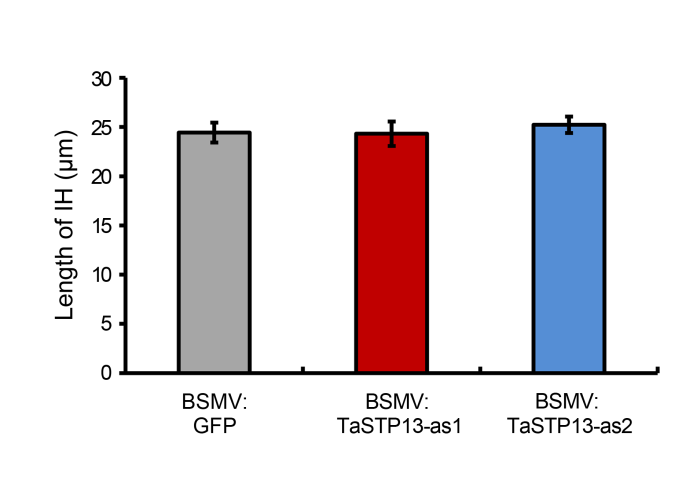


**Additional file 6. Figure S6. The length of IH in *TaSTP13*-silenced and control plants at 48 hpi.** No significant difference in the length of IH was observed between control and *TaSTP13*-silenced plants. The length of IH was measured from the substomatal vesicle to the apex of the longest infection hyphae. Values are represented as the mean ± SD of three independent samples with 50 infection sites each. Significance was determined using one-way ANOVA.
