# Supplementary material for: TaSTP13 contributes to wheat susceptibility to stripe rust possibly by increasing cytoplasmic hexose concentration
Source: BMC Plant Biol. 2020 Jan 30;20:49. doi: 10.1186/s12870-020-2248-2 (PMC6993525; doi:10.1186/s12870-020-2248-2)
Supplement: Supplementary file 7 — Additional file 7: Figure S7. RT-PCR analysis of TaSTP13 expression in TaSTP13-OE and wild type plants. AtUBC21 was used as the control (bottom panel). [file 12870_2020_2248_MOESM7_ESM.docx]

**

**

**Additional file 7. Figure S7.** **RT-PCR analysis of *TaSTP13* expression in TaSTP13-OE and wild type plants.** *AtUBC21* was used as the control (bottom panel).
