# Supplementary material for: TaSTP13 contributes to wheat susceptibility to stripe rust possibly by increasing cytoplasmic hexose concentration
Source: BMC Plant Biol. 2020 Jan 30;20:49. doi: 10.1186/s12870-020-2248-2 (PMC6993525; doi:10.1186/s12870-020-2248-2)
Supplement: Supplementary file 8 — Additional file 8: Table S1. DNA constructs in this study. [file 12870_2020_2248_MOESM8_ESM.docx]

Additional file 8. Table S1. DNA constructs in this study.

| Construct name | vector | Description |
| --- | --- | --- |
| pT-simple--*TaSTP13* | pMD 19-T vector | Cloning of *TaSTP13* |
| pK7FWG2-*TaSTP13* | pK7FWG2 | Overexpression of *TaSTP13* in *Arabidopsis* and *N. benthamiana* |
| TOPO-*TaSTP13* | pENTR™/D-TOPO® | The Gateway vector for subclone |
| BSMV:*GFP* | pBSMVγ | For BSMV negative control |
| BSMV:*TaPDS* | pBSMVγ | For BSMV positive control |
| BSMV:*TaSTP13*-as1 | pBSMVγ | For BSMV mediated silencing of *TaSTP13* |
| BSMV:*TaSTP13*-as2 | pBSMVγ |  |
| pTF486-*TaSTP13* | pTF486 | For TaSTP13 subcellular localization in wheat protoplast |
| pDR195-*TaSTP13* | pDR195 | Expression of TaSTP13 in yeast |
| pDR195-*GFP* | pDR195 | For subcellular localization in yeast |
| pDR195-*TaSTP13*-*GFP* | pDR195 |  |
| pBT3-N-*TaSTP13* | pBT3-N | For Split-Ubiquitin Analysis |
| pPR3N-NubG-*TaSTP13* | pPR3N |  |
| *TaSTP13*-pSPYNE(R)173 | pSPYNE(R)173 | For BiFC assay |
| *TaSTP13*-pSPYCE | pSPYCE(M) |  |
| *TaSGT1*-pSPYNE(R)173 | pSPYNE(R)173 | For BiFC positive control |
| *TaRAR1*-pSPYCE | pSPYCE(M) |  |
