# Supplementary material for: TaSTP13 contributes to wheat susceptibility to stripe rust possibly by increasing cytoplasmic hexose concentration
Source: BMC Plant Biol. 2020 Jan 30;20:49. doi: 10.1186/s12870-020-2248-2 (PMC6993525; doi:10.1186/s12870-020-2248-2)
Supplement: Supplementary file 9 — Additional file 9: Table S2. The primers and strains used in this study. [file 12870_2020_2248_MOESM9_ESM.docx]

**Additional file 9. Table S2. The primers and strains used in this study.**

| Primer name | Sequence | Annotation |
| --- | --- | --- |
| TaSTP13F | ATGCCGGGCGGGGGGTTCGCCGTGT | Amplification of ORF of *TaSTP13* |
| TaSTP13R | TCAGACGGTGGCGTTCTTGCCGTTG |  |
| qRT-PCR-TaSTP13ABDF | GTGGAAGCAGCACTGGTTCTGGAAG | Quantitative RT-PCR of *TaSTP13* transcripts |
| qRT-PCR-TaSTP13-4AR | GCACATCTTACACACCAGACGAGAT |  |
| qRT-PCR-TaSTP13-4BR | CTGCTGTTCCTCGTCGCATATGGAA |  |
| qRT-PCR-TaSTP13-4DR | CTCGCGACTGAAAGTGTCTGCACTC |  |
| TaSTP13-pBT3NF | tgcagggccattacggccATGCCGGGCGGGGGGTTCGC | Construction of split ubiquitin system |
| TaSTP13-pBT3NR | atgggggccaaagcggccTCAGACGGTGGCGTTCTTGC |  |
| TaSTP13-pPR3NF | gacgcgtggccattacggccATGCCGGGCGGGGGGTTCGCCG |  |
| TaSTP13-pPR3NR | ctcgagaggccgaggcggccGTCAGACGGTGGCGTTCTTGCCG |  |
| GFP-pDR195F | ccgctcgagATGGTGAGCAAGGGCGAGGA | Overexpression of *TaSTP13* in *S.*  *cerevisiae* |
| GFP-pDR195R | cgcggatccTTACTTGTACAGCTCGTCCATG |  |
| TaSTP13-pDR195F | ccgctcgagATGCCGGGCGGGGGG |  |
| TaSTP13-pDR195R | gaatgcggccgcTCAGACGGTGGCGTT |  |
| Overlapping-R | TGCAACAGGAACATCTGCACCACCAGCGTGGCCAGCACGT |  |
| Overlapping-F | ACGTGCTGGCCACGCTGGTGGTGCAGATGTTCCTGTTGCA |  |
| TaSTP13-pSPYNE(R)173 | ggactagtATGCCGGGCGGGGGG | Construction of BiFC Assay |
| TaSTP13-pSPYNE(R)173 | ccgctcgagGACGGTGGCGTTCTT |  |
| TaSTP13-pSPYCE | ggactagtATGCCGGGCGGGGGG |  |
| TaSTP13-pSPYCE | ccgctcgagGACGGTGGCGTTCTT |  |
| TaSTP13-VIGS-F1 | ccttaattaaTCCGGCGTGGAGTTCGAGGC | Construction of pBSMVγ: *TaSTP13* |
| TaSTP13-VIGS-R1 | aaatatgcggccgcAAGGTGGCAGTGAGGCCGGC |  |
| TaSTP13-VIGS-F2 | ccttaattaaGGGCAGAGCGTGACGGTGTG |  |
| TaSTP13-VIGS-R2 | aaatatgcggccgcCTTCCAGAACCAGTGCTGCTT |  |
| GFP-VIGS-F | ccttaattaaTCACCTACGGCGTGCAGTGC | Construction of pBSMVγ: *GFP* |
| GFP-VIGS-R | aaatatgcggccgcTCGAACTTCACCTCGGCGCG |  |
| TaSTP13-486F | acgcgtcgacATGCCGGGCGGGGGG | For TaSTP13 subcellular localization in wheat protoplast |
| TaSTP13-486R | atttgcggccgcAGACGGTGGCGTTCTT |  |
| TaSTP13-pk7F | caccATGCCGGGCGGGGGG | Overexpression of *TaSTP13* in *Arabidopsis* and TaSTP13 subcellular localization in *N. benthamiana* |
| TaSTP13-pk7R | GACGGTGGCGTTCTTGCCGT |  |
| TaEF-F | TGGTGTCATCAAGCCTGGTATGGT | Internal reference of qRT-PCR |
| TaEF-R | ACTCATGGTGCATCTCAACGGACT |  |
| PstEF-F | TTCGCCGTCCGTGATATGAACAA |  |
| PstEF-R | ATGCGTATCATGGTGGTGGAGTGA |  |
| Check-TaSTP13-F | GGGCAGAGCGTGACGGTGTGCGTCA | RT-PCR of *TaSTP13* in transgenic lines |
| GFPcheck-R | CAGGGTCAGCTTGCCGTAGGTGGCA |  |
| AtUBC21F | TCATAGCATTGATGGCTCATCCT | PCR analysis of *Arabidopsis* line |
| AtUBC21F | ACCCTCTCACATCACCAGATCTTAG |  |
| Strains | Genotype/comment | Apllication |
| JM109 | *E. coli*; recA1 , supE44, endA1, hsdR17, gyrA96, relA1, thi, Δ(lac-proAB) | Constructions of the plasmids |
| GV3101 | *A. tumefaciens*; Rif^r^, Gen^r^ | Transient expression of *TaSTP13* |
| DH5α | *E. coli*; F- 80lacZ△M15△, (lacZYA-argF), U169, endA1, recA1hsdR17(rk-,mk+) supE44λ- thi-1, gyrA96, relA1, phoA | Constructions of the plasmids |
| EBY.VW4000 | EBY.VW1000 stl1v : :loxP agt1v : :loxP ydl247wv: :loxP yjr160cv: :loxP | Functional identification of *TaSTP13* |
| NMY51 | MATa his3200 trp1-901 leu2-3,112 ade2 LYS2::(lexAop)4-HIS3 ura3::(lexAop)8-lacZ ade2::(lexAop)8-ADE2 GAL4 | Yeast reporter strain for split ubiquitin system |

*Uppercase letters indicate bases that match the initial template.

Lower case letters indicate 5′ extensions that do not match the initial template. Restriction sites introduced into the amplicons are underlined.
